# A theoretical framework of immune cell phenotypic classification and discovery

**DOI:** 10.3389/fimmu.2023.1128423

**Published:** 2023-03-02

**Authors:** Yuzhe Hu, Chen Liu, Wenling Han, Pingzhang Wang

**Affiliations:** ^1^ Department of Immunology, NHC Key Laboratory of Medical Immunology (Peking University), School of Basic Medical Sciences, Peking University Health Science Center, Beijing, China; ^2^ Peking University Center for Human Disease Genomics, Beijing, China; ^3^ Department of Clinical Laboratory, Peking University People’s Hospital, Beijing, China

**Keywords:** gene plasticity, plasticity-based classification, plasticitome, plasticitomics, immunophenotype, parathymosin, SPINK2, CDHR1

## Abstract

Immune cells are highly heterogeneous and show diverse phenotypes, but the underlying mechanism remains to be elucidated. In this study, we proposed a theoretical framework for immune cell phenotypic classification based on gene plasticity, which herein refers to expressional change or variability in response to conditions. The system contains two core points. One is that the functional subsets of immune cells can be further divided into subdivisions based on their highly plastic genes, and the other is that loss of phenotype accompanies gain of phenotype during phenotypic conversion. The first point suggests phenotypic stratification or layerability according to gene plasticity, while the second point reveals expressional compatibility and mutual exclusion during the change in gene plasticity states. Abundant transcriptome data analysis in this study from both microarray and RNA sequencing in human CD4 and CD8 single-positive T cells, B cells, natural killer cells and monocytes supports the logical rationality and generality, as well as expansibility, across immune cells. A collection of thousands of known immunophenotypes reported in the literature further supports that highly plastic genes play an important role in maintaining immune cell phenotypes and reveals that the current classification model is compatible with the traditionally defined functional subsets. The system provides a new perspective to understand the characteristics of dynamic, diversified immune cell phenotypes and intrinsic regulation in the immune system. Moreover, the current substantial results based on plasticitomics analysis of bulk and single-cell sequencing data provide a useful resource for big-data–driven experimental studies and knowledge discoveries.

## Introduction

Immune cells are highly heterogeneous in phenotypes. This heterogeneity is reflected not only in the different immune cell types from the perspective of lineage development but also in the same cell types that comprise multiple functional subsets, such as Th1, Th2, Th9, Th17, Th22 and follicular helper T (Tfh) cells characterized by a specific cytokine profile in CD4^+^ T cells (1). It is similar for CD8^+^ T cells (cytotoxic T cells/Tcs) since multiple effector subsets mirroring CD4^+^ T helper subsets have been described, including conventional IFN-γ-producing Tc1s, interleukin (IL)-4-producing Tc2s, IL-9-producing Tc9s, IL-17-producing Tc17s, and IL-22-producing Tc22s (2). From the perspective of immune regulation, in addition to classic CD4^+^ Treg cells, there are multiple other subsets with regulatory functions, such as CD8^+^ Treg cells (3), regulatory B cells (Bregs) (4), regulatory DCs (DCregs) (5), and regulatory innate lymphoid cells (ILCregs) (6).

It is not merely T cells that show high heterogeneity (7, 8), but nearly all the main immune cell types, such as B cells (9, 10), monocytes (11), macrophages (12, 13), DCs (14), NK cells and ILCs (15), are highly heterogeneous. The heterogeneity of these cells represents multiple functional cell states, which are associated with diversified immunophenotypes. However, what is the mechanism behind this heterogeneity? How is it determined? On the other hand, different functional subsets are capable of conversion (e.g., the transitions from Th17 or Th2 to Th1 or from Tregs or Tfh to Th17) under certain conditions, which suggests phenotypic plasticity or flexibility (16, 17). Therefore, are there common constraint mechanisms or some undiscovered rules in the process of phenotypic conversion of immune cells?

The existing classification system of immune cells focuses on the developmental relationship between lineages, which cannot effectively explain the diversified immune cell phenotypes and the phenotypic transition, nor can it predict novel immune cell phenotypes relevant to specific functional cell states. The high heterogeneity of immune cell phenotypes has even led to a dispute about the nomenclature of Th-cell subsets (18). The current challenge for immunologists is to find a phenotypic classification not only for Th cells (19) but also for other immune cells. Therefore, it is necessary to establish a novel theoretical system or framework that can explain the diversity of immune cells, phenotypic plasticity and conversion, and underlying phenotypic restrictiveness. Most importantly, the novel system should not contradict the existing classification system; in addition, it should have enough inclusive characteristics, that is, it should be open and compatible with novel immune cell subsets discovered in the future.

In this study, we proposed a novel framework to classify immune cells based on gene plasticity. In this study, immune cell subsets mainly refer to functional subsets, which are associated with specific functional cell states defined by various immunophenotypes. The system includes two core viewpoints: (1) immune cell subsets can be divided into subdivisions based on their highly plastic genes; (2) we pay less attention to the developmental relationships between immune cells but emphasize that the gain of phenotype accompanies the loss of phenotype during phenotypic change. Gene plasticity refers to the change in gene expression in response to conditions (20). It reflects the dynamic change and expressional variability. It is quantitatively measured by the gene plasticity (GPL) score when considering the change in the amount of mRNA and the resulting changes in the rank percentile values (20, 21). Highly plastic genes present a broad range of expression levels across samples; for example, they show high expression in some conditions but no or low expression under other conditions. However, lowly plastic genes generally show widely high or low/no expression across conditions. Highly plastic genes were previously shown to be suitable for marker molecules to label immune cell subpopulations (21). However, our previous study mainly focused on Th cells and did not elaborate a novel classification system in a broad sense.

To elaborate the current system, in this study, both RNA sequencing (RNA-Seq) and microarray data from human T cells, B cells, natural killer cells (NKs) and monocytes were collected and used for gene plasticity analysis. We optimized the quantitative evaluation method of gene plasticity and measured gene plasticity for over 16,500 genes shared by both sequencing and array platforms. Then, we collected and summarized thousands of immunophenotypes reported in the literature and found that highly plastic genes play an important role in maintaining immune cell phenotypes, suggesting that the current classification model should be compatible with the traditionally defined functional subsets.

The current model explains the infinite diversity of immune cells. Through correlated and anticorrelated gene analysis *via* virtual sorting (21, 22), the acquired (or cophenotypes) and lost (or mutually exclusive phenotypes) phenotypes related to highly plastic genes were also analyzed. The single-cell transcriptome supported the reliability of the results. The current gene plasticity model also effectively predicts novel immune cell subsets, such as SPINK2 and CDHR1 single-positive NKs, both of which are implied to play an important role in maternal-fetal interactions based on single-cell transcriptomic data, irrespective of their differential phenotypes.

Therefore, the system provides a new perspective of the characteristics of dynamic, diversity, layerability and intrinsic regulation mechanisms, as well as the functional clues, of immune cells. It is simple in concept and easy to connect with the known concept of cell plasticity. Moreover, the large number of omics results in this study will contribute to the discovery of novel functional subsets and their regulation.

## Materials and methods

### Datasets and bulk transcriptomic data analysis

Microarray datasets from the Affymetrix Human Genome U133 Plus 2.0 Array were directly from our previous reports (20–22) and updated to incorporate the latest samples. The raw data were downloaded from the Gene Expression Omnibus (GEO, https://www.ncbi.nlm.nih.gov/geo/) (23) and uniformly processed as described previously (20, 24). We focused on human CD4^+^ and CD8^+^ T cells, B cells, NKs and monocytes in the current analysis. The percentile rank scores of genes were calculated based on their expressional signal values within each array (20, 21). Briefly, the signal values were first sorted from smallest to largest and divided into 100 equal parts with successive values from 1 to 100, which were used for the percentile rank scores for the genes in each part. By default, the rank score means the percentage rank score in this study. The probe set with the maximum expression level was selected when a gene had multiple probe sets.

Bulk RNA-Seq datasets from high-throughput sequencing (HTS) were downloaded from the Sequence Read Archive (SRA) database (25). We applied NCBI’s SRA Toolkit to download and convert SRA files to FASTQ files. Then, we applied FastQC (http://www.bioinformatics.babraham.ac.uk/projects/fastqc/) to view the read quality, used the STAR_2.5.3a (26) tool to align reads to the human genome (hg19), and applied featureCounts v1.6.0 (27) to calculate raw read counts at the gene level. All analyses were performed based on the standard pipelines as described in the corresponding tool manuals. Quality control was performed in each sample as follows: the total read count of protein-encoding genes was larger than or equal to 500,000; protein-encoding genes accounted for more than 50% of the total read count of all genes; and the number of detected protein-encoding genes was larger than or equal to 10,000. In addition, when a sample had multiple run data, although it was not very common, the SRA file containing the largest number of genes was selected. Protein-encoding genes were extracted from raw count files to calculate their TPM (transcripts per million) values. Then, percentile rank scores, which range from 0 to 100, were converted directly from TPM values and were calculated based on the formula: P = n/N*100, where P = percentile rank score, N = number of nonzero TPM values, and n = ordinal rank of a gene, and the TPM values of all genes were in ascending order in each assay. When a gene’s read count equals zero, its percentile rank score was set to 0.

For both microarray and RNA-Seq data, quality control was also performed based on marker gene expression. Marker genes were selected as follows: CD79A and CD79B for B cells; CD3D, CD3G and CD4 for CD4^+^ T cells; CD3D, CD3G, CD8A and CD8B for CD8^+^ T cells; GNLY (granulysin) and NKG7 (natural killer cell granule protein 7) for NKs; and CD14 for monocytes (20, 28, 29).

### Calculation of absolute gene plasticity score

We previously used the gene plasticity (GPL) score to quantitatively measure gene plasticity, with larger scores indicating more variability or higher plasticity in gene expression (20). The average rank score (ARS) of a gene was the mean of the percentile rank scores in all samples and represented a gene’s average expression level across various conditions (20). The formula for the ARS calculation was:

$ARS=\frac{\sum_{i=1}^{n}P_{i}}{n}$ , where *P* indicates the percentile rank score and n is the number of samples. According to the distribution of rank scores, the interquartile range (IQR) was defined as the difference between the 1st quartile (25th percentile, 1st Qu, Q1) and 3rd quartile (75th percentile, 3rd Qu, Q3) of expressional percentile rank scores and used to measure gene plasticity as the GPL score (IQR method) (20–22), that is, GPL score (iqr) = IQR = Q3 – Q1. In this study, however, the absolute GPL score was also used to measure gene plasticity. This was the difference between the maximum and minimum percentile rank scores in the samples, that is, GPL score (absolute) = P (max) – P (min), where P indicated percentile rank score. Therefore, an absolute GPL reflects an absolute level of expressional plasticity, and a larger absolute GPL score indicates higher plasticity.

### Collection of known immunophenotypes and marker gene annotation

To systematically collect immune cell subsets in the literature, publications from 56 journals related to immunology, such as *Nature Review Immunology*, *Annual Review of Immunology* and *Nature Immunology*, were downloaded. In addition, PubMed at NCBI was searched by querying key words ‘(subset OR subpopulation) AND (T cells OR Treg OR B cells OR Breg OR plasma cells OR natural killer cells OR natural killer T cells OR monocytes OR macrophages OR dendritic cells OR innate lymphoid cells OR neutrophils OR granulocytes)’. The retrieved result in the NBIB (NCBI PubMed Export File) file was used as input to the reference managing software EndNote (https://www.endnote.com/) to obtain the full text in PDF (Portable Document Format) file format through an automatic built-in download function of the tool. Then, all PDF files were converted to text format files using the command ‘pdftotext’ developed by Poppler Developers (http://poppler.freedesktop.org).

The phenotypes of immune cell subsets are generally described as a form comprising both marker molecules and expression labels (often as superscripts), such as ‘CD45RA^+^CCR7^-^’ T cells, where the plus sign indicates positive expression while the negative sign indicates no expression, and this immunophenotype generally refers to the protein level. Therefore, the ‘grep’ command under a Linux operation system was used to extract all possible immunophenotypes in the text format publications by regular expression patterns, such as ‘.{0,20}[+−].{0,12}[TB].{0,12}cell’. In the pattern, the dot indicates any character, the numbers in curly braces indicate the minimum and maximum repeat times, and the characters specified within square braces indicate any matching could match the majority of T and B-cell phenotypes. In addition, expression labels, such as ‘lo’, ‘bright’, ‘dim’, ‘int’, ‘medium’, ‘mid’, ‘high’ and ‘hi’, were also taken into consideration during pattern matching. The retrieved immunophenotypes were further deduplicated and checked for species sources.

Marker molecules extracted from immunophenotypes are often not the standard gene symbols. Therefore, we converted all of the aliases to the standard Entrez gene symbols (30). These standardized immunophenotypes were further deduplicated irrespective of the writing order of marker genes. After conversion, a nonredundant gene set was obtained and used to perform subsequent analysis.

### Functional enrichment analysis of gene sets

Functional annotations of marker gene sets were performed using the online DAVID database (2021 update) (31), which incorporates a series of functional annotation categories, such as Gene Ontology (GO), KEGG (Kyoto Encyclopedia of Genes and Genomes) pathways, UniProtKB Keywords (UP_KW) protein domains and interactions. GO depicts three main biological concepts: biological process (BP), molecular function (MF) and cellular component (CC). The enrichment with annotation terms with *p* value ≤ 0.05, as well as the adjusted *p* values, was selected and shown based on the online results (31).

Gene set enrichment analysis (GSEA) is an enrichment analysis method used to determine whether members of a gene set S tend to occur toward the top (or bottom) of list L (32). We made a list of retrieved marker genes from known immune cell subsets as the gene set S and compared them with the list of all plastic genes ranked by absolute GPL score (in descending order) as the list L in each cell type. The GSEAPreranked module was used to run GSEA. Herein, it determines whether the marker gene set shows statistically significant enrichment at either end of the ranked gene set, that is, how often members in the marker gene set occur at the extremely high (top of the list) or low (bottom of the list) plastic genes.

### Virtual sorting analysis

Virtual sorting (or *in silico* sorting) means analyzing a large number of data samples in a virtual way because there are no actual immune cells sorted as done in the FACS process (21, 22). It was used to select cell samples according to gene expression intensity, which was similar to the gating process in flow cytometry, to perform a series of analyses (20–22). In this study, we conducted virtual sorting to identify the correlated and anticorrelated genes of a highly plastic gene; however, in contrast to previous studies (21, 22), the k-means (k = 2) method but not quartiles was used to divide samples into two groups based on the differential expression states of a highly plastic gene. The difference ARSs, namely, the delta (δ) value, of all genes in the two groups were calculated using the formula δ_i_ = ARS_i(g1)_-ARS_i(g2)_, where *i* indicates the *i* ^th^ gene in both groups with the same gene order. For a highly plastic gene, the positive or high group was the first group (g1), whereas the negative or low group was the second group (g2). Delta values of all genes were sorted in descending order and the virtual sorting results were further filtered with Pearson correlation coefficients larger than or equal to 0.5 for the correlated genes, whereas the results were filtered with Pearson correlation coefficients less than 0 and cosine similarity less than or equal to 0.1 for the anticorrelated genes. In this study, the cosine similarity varied from 0 to 1. When two vectors (expressional values of paired genes) had the same orientation, the angle between them was 0, and the cosine similarity was 1; however, when two vectors had a 90-degree angle between them and a cosine similarity of 0.

### Coexistence and mutually exclusive rate analysis

For microarray, the coexistence rate between two or paired genes across samples was calculated based on the formula: coexistence rate = (C_PP_+C_AA_)/N, whereas C_PP_ and C_AA_ indicated sample counts with both (present-present, PP) or neither (absent-absent, AA) of the genes’ expression within the same samples according to the detection call (24) and N was the total sample number. The formula for the APA (absent-present and present-absent) rate was APA rate = 1 - coexistence rate. Therefore, the APA rate represented the degree of mutually exclusive expression as described in our previous study (21). For RNA-Seq data, a series of cutoffs of TPM values including 1, 2, 3, 4, 5, 10 and 20 were tested to evaluate coexistence or mutual exclusion. For example, when the cutoff was set to 5, PP meant both genes had TPM values larger than or equal to 5, whereas AA meant both genes’ TPM values were less than 5.

### Single-cell RNA-Seq analysis

Single-cell datasets were mainly from two resources, the GEO database and UCSC Cell Browser (http://www.cells.ucsc.edu/) (33). For datasets from GEO, a standard analysis pipeline using the R Seurat package (version 3.1.2) (34) was used for single-cell analysis. Cell types were annotated based on the labels directly from data submitters or according to marker gene expression. Briefly, the raw count of genes in each cell was first normalized (unless a normalized expression matrix was provided by the data submitter), and then the top 2,000 highly variable genes (HVGs) were selected. After the data were scaled and centered, principal component analysis (PCA) based on HVGs was conducted. For dimensionality reduction for single-cell cluster visualization, the uniform manifold approximation and projection (UMAP) method was used. Cell type labels were directly from the authors’ annotation. When there was no annotation, cell type-specific marker genes, either from typical well-known markers or directly from the respective literature, were used to confirm cell types, such as CD3D and CD4 for CD4^+^ T cells, CD8A and CD8B for CD8^+^ T cells, CD79A for B cells, CD14 for monocytes and NKG7 for NKs (28, 29). For datasets from the UCSC Cell Browser, the website provides a gene expression matrix, cell meta annotation and dimensionality reduction coordinates for each dataset; therefore, immune cells could be directly extracted and annotated based on this known information.

### Isolation of human peripheral blood mononuclear cells

The anticoagulant whole blood samples of healthy people in this study were obtained from Peking University People’s Hospital. Sample collection was given informed consent by providers and approved by the Peking University Biomedical Ethics Committee. Fresh anticoagulation whole blood was diluted with an equivolume of RPMI 1640 medium, and the diluted blood was spread above the separating medium. The interface was kept clear and then centrifuged at 500 g for 20 minutes at room temperature (brake adjusted to 0). After centrifugation, the lymphocyte layer (the white layer) was carefully absorbed and washed 3 times at 4°C. Then the lymphocytes were stained for flow cytometry or cultured in RPMI 1640 medium with 10% heat-inactivated fetal bovine serum (FBS) and 1% penicillin streptomycin mixture (PS). For the lymphocyte activation test, the cultured lymphocytes were activated by PMA and ionomycin mixture (BioLegend, 423301, 1:500) stimulation for 8 h, 16 h and 24 h before expressional detection by flow cytometry (see below).

### Flow cytometry

The flow cytometry antibodies used in this study were as follows: anti-human CD3-APC-Cy7 (BD, 557832); anti-human CD4-BV510 (BD, 562970); anti-human CD8-PE-Cy7 (BD, 557746); anti-human CD45RA-BV605 (BD, 562886); anti-human CCR7-APC-R700 (BD, 565867); anti-human-CD8-APC (BioLegend, 300912); anti-human-CD25-PE (BioLegend, 302605); anti-human CD26-PE (BioLegend, 302705); anti-human CD49f-FITC (BioLegend, 313605); and the fluorescent dye 7-AAD (BioLegend, 420404). The cells were incubated with 1% Fc receptor blocker for 10 minutes and incubated with antibodies for 30 minutes. Flow cytometry analyses were conducted using the BD FACS Canto system. The data were analyzed with FlowJo software.

## Results

### A proposed model for phenotypic classification of immune cells based on gene plasticity

**Figure 1:**
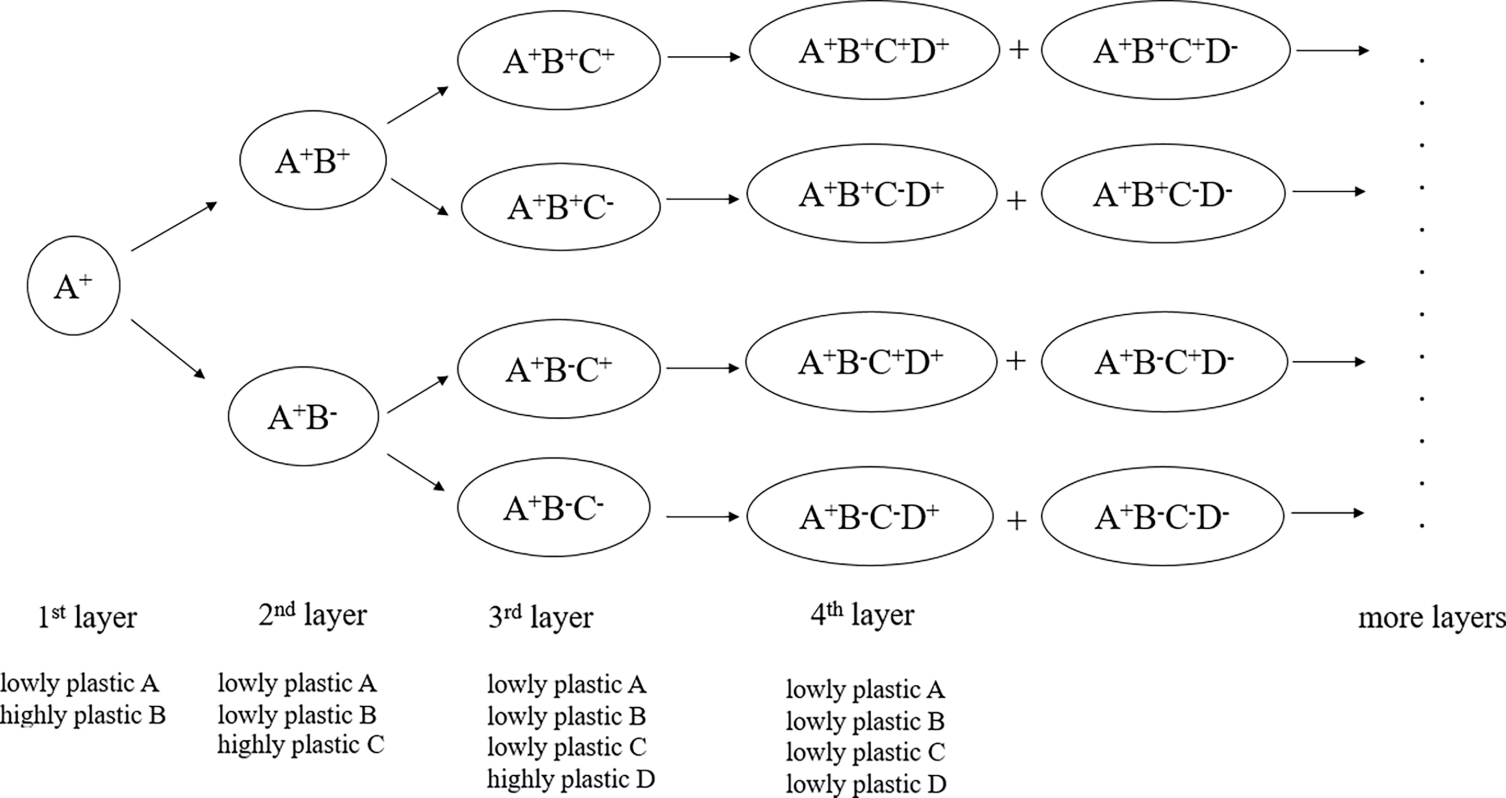
Gene plasticity model in the phenotypic classification and discovery of immune cell subsets. It represents a theoretical framework for subtle subdivisions of immune cell subpopulations based on gene plasticity. The core idea of the model is that small functional subsets can be divided from their greater subsets so that immune phenotypes can be continuously defined according to highly plastic genes, accompanied by gene plasticity changes in the marker genes. Therefore, phenotypic stratification can be conducted *via* the addition of highly plastic genes in each of the greater layers. During the process, the cophenotypes and mutually exclusive phenotypes related to these highly plastic genes are accompanied.

Immune cell phenotypes are generally expressed by marker genes and their combinations, which constitute immunophenotypes. In experiments, marker molecules are also used to label, identify and isolate immune cells. Different functional subsets, which are relevant to specific biological significance, are commonly distinguished and illustrated in different cell clusters based on the determination of the level of expression of fluorescently labeled marker molecules. Our previous studies revealed that gene plasticity could be used for marker evaluation and discovery of novel immune cell subpopulations (20, 21). These previous studies on gene plasticity, marker evaluation and immune cell subpopulation prediction prompted us to propose a model of immune cell phenotypic classification, as shown in **Figure 1**. This model could be called the gene plasticity model because the phenotypic classification was based on gene plasticity. It could also be called the ‘3s’ model because it represents subtle immune cell subdivisions of the initial subpopulations based on a series of successive highly plastic genes.

The system included two core points. One was that immune cell subsets associated with specific immunophenotypes could be divided into subdivisions based on their highly plastic genes when they existed. For example, as shown in **Figure 1**, among the initial cells with high expression of A, which indicated that A was low plastic in A^+^ cells, the highly plastic gene B in A^+^ cells produced phenotypes such as A^+^B^+^ and A^+^B^-^, which represented cell states with high and low/no expression of B, respectively. Similarly, each of an additional highly plastic gene, such as C and further D, would produce novel phenotypes represented by these genes and their combinations, making multiple subdivisions of the greater layers. As inferred by the model, when a highly plastic gene became the marker gene to label cell subpopulations, its plasticity decreased. This was because marker genes generally showed a broad range of either high or low expression levels in the relevant cell populations and in their descendant subdivisions.

For example, based on highly plastic genes, CD3^+^ T cells (layer 1) were divided into CD3^+^CD4^+^ and CD3^+^CD4^-^ T cells based on the marker CD4 molecule (layer 2). When FOXP3 was introduced (layer 3), the highly expressed FOXP3 in CD3^+^CD4^+^ cells could label Treg cells. The fourth layer comprises highly plastic genes in Tregs that further produce Treg subsets, such as CXCR3^+^ Tregs, CCR6^+^ Tregs, LAG3^+^ Tregs, TIM3^+^ Tregs and HLA-DRA^+^ Tregs (21). More subdivisions would be expected among these Treg subsets when their highly plastic genes were identified.

The other core point was that the loss of phenotype was accompanied by the gain of phenotype. In this model, we did not consider the sequence of genes and paid less attention to the developmental relationships among these cell subdivisions. The initial layer could be determined according to specific application scenarios in practice; therefore, there was no need to indicate that the first layer must start with an exact gene. However, we were concerned that when a layer was added to an existing phenotype, what phenotypes were destined to be lost (that is, the mutually exclusive phenotypes). This meant that when a highly plastic gene tended to be highly expressed (‘the gain of phenotype’) in response to conditions, a set of other genes tended to be coexpressed (‘cophenotype’) or inevitably lost expression (‘the loss of phenotype’).

The model did not mean that all involved functional cell states could be observed under only one or only limited conditions. It represented a comprehensive framework and a scenario that considered all possible conditions, such as individuals, tissues, induction or stimulation, disease states, and even development stages. This was because gene plasticity analysis represented condition-associated stratification. With the increase in experimental conditions, gene plasticity would also increase, at least for some genes, causing some specific phenotypes to be observable. On the other hand, the model also suggested that a lowly plastic gene would become highly plastic when it was placed in a greater layer. Considering the numerous highly plastic genes under various conditions, immune cell phenotypes should be continuously dividable and constitute infinite diversity. The following analysis explained and supported the logical rationality of the current model from multiple aspects.

### Genes show generally high plasticity revealed by either bulk RNA sequencing or microarray data

Our previous gene plasticity analysis was performed based on microarray data (20–22). In recent years, RNA sequencing (RNA-Seq) data have rapidly accumulated and can be made publicly accessible. RNA-Seq technology has several advantages over hybridization-based techniques, such as improved specificity and sensitivity and increased dynamic range in gene expression measurement. In this study, for the first time, we used RNA sequencing (RNA-Seq) data for gene plasticity analysis, which also facilitated comparison in various platforms. Therefore, we compiled two sets of datasets from human CD4^+^ T cells, CD8^+^ T cells, B cells, NKs and monocytes from both microarray (updated in this study) and bulk RNA-Seq data. After quality control, we finally obtained 2,468 microarray samples, which covered 20,283 nonredundant genes, from 632 (CD4^+^ T cells), 415 (CD8^+^ T cells), 503 (B cells), 149 (NKs) and 769 (monocytes) samples of the indicated cell types. In addition, 3,029 bulk RNA-Seq samples, including 768 (CD4^+^ T cells), 620 (CD8^+^ T cells), 377 (B cells), 204 (NKs) and 1060 (monocytes) samples, were finally processed to generate TPM values of 20,589 protein-encoding genes. These data were used for subsequent gene plasticity analysis.

**Figure 2:**
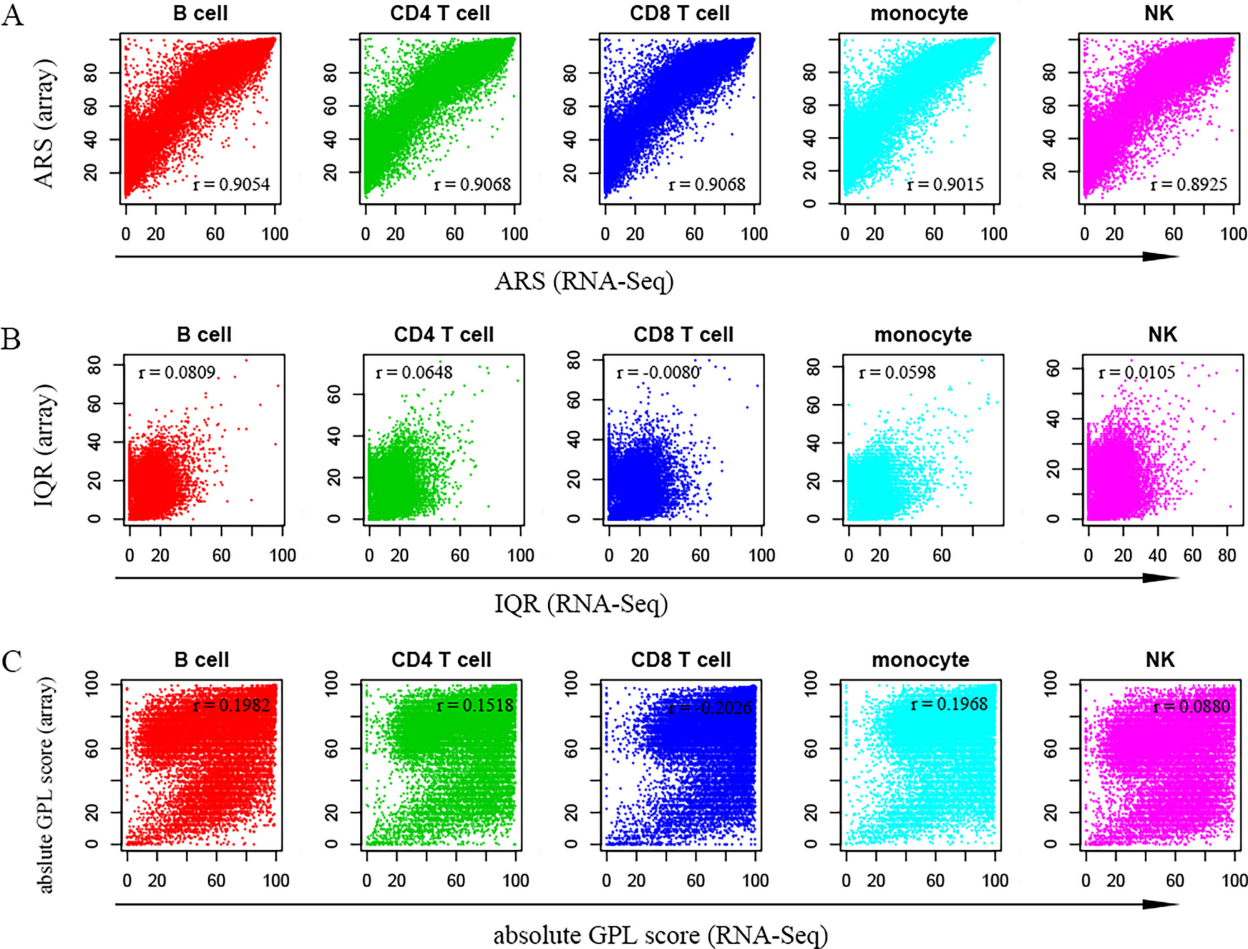
Scatter plots show the relationships between measured values calculated using RNA-Seq and microarray datasets. **(A–C)** represent the average rank score (ARS), interquartile range (IQR) and absolute GPL score, respectively. In each panel, the x-axis indicates values from RNA-Seq data, while the y-axis indicates values from microarray data. The Pearson coefficient (r) is shown in each panel. Therefore, there is a high linear correlation between the ARSs of the two platforms.

As shown in **Figure 2A**, the average rank scores (ARSs) derived from both platforms were highly positively correlated with Pearson correlation coefficients of 0.9054 (B cells), 0.9068 (CD4^+^ T cells), 0.9068 (CD8^+^ T cells), 0.9015 (monocytes), and 0.8925 (NKs) in the respective cell types. This suggested that the data from the two platforms (microarray & RNA-Seq) should be comparable after percentile rank normalization. However, the overall distribution of ARSs from the microarray was above that from RNA-Seq (**Figure 2A**). In addition, when the common genes were extracted in both platforms to recalculate their ARSs, an overall higher distribution of ARSs from the microarray datasets was also observed, although the correlation strength was further improved (**Figure S1**). This suggested decreased noise signals in the RNA sequencing data. For example, the ARSs of EEF1G (eukaryotic translation elongation factor 1 gamma) were larger than 99 in all five cell types from the array; however, the ARSs were limited to no more than 2 in these cells from RNA-Seq (**Table S1**). Similar results were also observed for LUC7L2 (LUC7-like 2, premRNA splicing factor), PDE4C (phosphodiesterase 4C), and PIGY (phosphatidylinositol glycan anchor biosynthesis class Y) (**Table S1**). Therefore, the high expression of these genes in the microarray may result from a high noise signal caused by nonspecific binding in the process of DNA hybridization.

For the microarray, the average absolute GPL scores of all genes in the five cell types were 62.88 (B cells), 65.31 (CD4^+^ T cells), 64.75 (CD8^+^ T cells), 66.71 (monocytes) and 56.01 (NKs), whereas for RNA-Seq, the average absolute GPL scores were 57.52 (B cells), 63.89 (CD4^+^ T cells), 71.42 (CD8^+^ T cells), 67.29 (monocytes) and 56.26 (NKs). Considering that the maximum absolute GPL score was 100, the scores from both platforms revealed that genes generally showed high plasticity. However, in contrast to the ARS, there was no apparent linear correlation in either quartile GPL scores (**Figure 2B**) or the absolute GPL scores (**Figure 2C**). This may be explained by the independent samples related to various conditions observed in different platforms.

A list of 16,513 genes and the associated ARSs and GPL scores identified in both platforms are shown in **Table S1**, which provides a quick evaluation of gene plasticity and average gene expression levels.

### Highly plastic genes play an important role in defining immune cell subsets and maintaining immune cell phenotypes

To investigate whether the existing immune cell subsets conform to the current gene plasticity model, we first tried to collect known functional subsets reported in the literature, extracted the marker genes from the immunophenotypes and evaluated their expressional gene plasticity. In total, approximately 170,000 papers from 56 immunological journals were analyzed. This made a total of 4,990 subsets with nonredundant immunophenotypes, including 2,757 human and 2,233 mouse subsets, from all the main immune cell types, such as T cells (including Treg cells and other T cells), B cells, plasma cells, NKs, natural killer T (NKT) cells, monocytes, macrophages, dendritic cells (DCs), innate lymphoid cells (ILCs) and granulocytes (including neutrophils, eosinophils, basophils and other granulocytes). These subsets represented functional subsets related to specific functional cell states, which were generally gated by FACS technology.

We mainly focused on human CD4^+^ T cells, CD8^+^ T cells, B cells, NK cells and monocytes, which were the most abundant among the collected human immunophenotypes (**Table S2**). In total, we obtained 1,529 (T cells), 414 (B cells), 257 (NKs) and 141 (monocytes) immunophenotypes for the indicated cell types (**Table S2**). Among T cells, 830 and 379 immunophenotypes were directly annotated as CD4 and CD8 single-positive T cells, respectively. These immunophenotypes involved 171 (CD4^+^ T cells), 122 (CD8^+^ T cells), 116 (B cells), 104 (NKs) and 83 (monocytes) nonredundant marker genes in the respective cells (**Table S2**).

**Figure 3:**
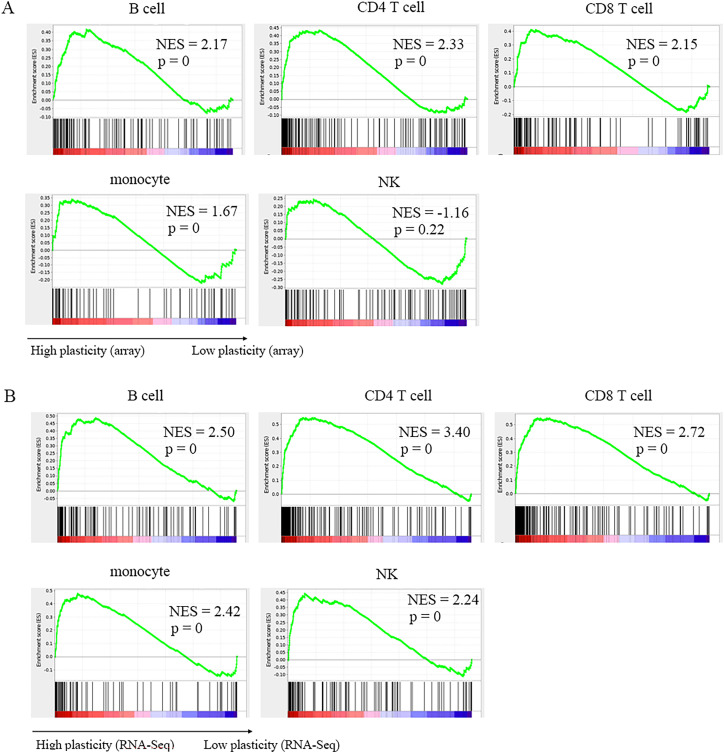
Functional enrichment analysis of marker gene sets based on gene plasticity measured by absolute GPL score. **(A, B)** represent results from microarray **(A)** or RNA-Seq **(B)** data in the indicated cell types.

Gene set enrichment analysis (GSEA) represents an efficient and unbiased evaluation of the gene plasticity of these known marker genes. As shown in **Figure 3**, marker genes extracted from CD4^+^ T cells, CD8^+^ T cells, B cells and monocytes were significantly enriched in the highly plastic gene regions from either microarray or RNA-Seq. Significant enrichment of marker genes was also observed in NKs from RNA-Seq but not in NKs from microarrays (**Figure 3**). We considered that this could result from the lowest sample size of NKs in the microarray data because limited conditions affected the observed gene plasticity states of the marker genes. We also found that the normalized enrichment score (NES) from RNA-Seq data in each cell type was higher than that from the microarray (**Figure 3**), suggesting that RNA-Seq data offer improved gene plasticity evaluation. We found that NES values were positively correlated with the marker gene numbers, suggesting that when there were enough subsets identified, the marker genes would become more aggregated in highly plastic genes.

However, there was minor aggregation in lowly plastic gene regions (**Figure 3**). This was because some marker genes, although they were contained in immunophenotypes, were widely either expressed or not expressed in the cell types and showed relatively low plasticity. For example, lineage marker genes, such as CD19, CD20 (also known as MS4A1/membrane spanning 4 domains A1) and CD79A in B cells, showed extensively high expression and low plasticity in B cells (**Table S2**). In addition, because the current immunophenotypes mainly represented the protein level, it was not excluded that some marker genes might be highly plastic at the protein level but lowly plastic at the RNA level. It was also possible that the currently limited samples and conditions led to high plasticity states not observed for some genes. The sparse occurrence of marker genes in the moderately plastic regions in the cells (**Figure 3**), such as CD4^+^ T cells, CD8^+^ T cells and monocytes, further confirmed that marker genes were generally highly plastic.

Therefore, the global analysis further supported that highly plastic genes were suitable to label immune cell subpopulations (21). In addition, this result revealed a key role of highly plastic genes in defining functional subsets and maintaining immunophenotypes. For example, based on the absolute GPL scores from either microarray or RNA-Seq data, the characteristic cytokines for Th1 (IFNG), Th2 (IL4, IL5, IL13), Th17 (IL17A, IL17F, IL22, IL26), Th9 (IL9), Th22 (IL22), Tfh cells (IL4, IL21) and Tregs (IL10, TGFB1) were extremely plastic in CD4^+^ T cells (**Table S1**) (1, 16, 21). In addition, master transcription factors, such as TBX21 (T-box transcription factor 21, also known as T-bet) for Th1 cells, RORC (retinoic acid receptor-related orphan receptor gamma, also known as RORγt) for Th17 cells, FOXP3 (forkhead box protein P3) for Tregs, and BCL6 (B-cell lymphoma 6/BCL6 transcriptional repressor) for Tfh cells, were also highly plastic (**Table S1**) (1, 16, 21). More examples of extremely plastic genes as markers of known immune cell subsets in CD4^+^ T cells, CD8^+^ T cells, NKs, B cells and monocytes are shown in **Figure S2** and **Table S2** (see below).

### Known functional subsets are compatible with the gene plasticity model and can be further subdivided

In the classification model, the high plasticity genes used as markers were layered. This model did not consider the order of marker genes, and any single marker gene could be used as the first layer, which represented the marker gene itself. From the second layer, immune cell subdivision represented a combination of marker genes. To systematically explain the role of highly plastic genes in known phenotypes, we divided the immunophenotype into an immunophenotypic unit, which was composed of the marker gene and its expression tag, which was used to explain the expression intensity. Therefore, each known parent immunophenotype with more than one layer could be split into several nonredundant child immunophenotypes, irrespective of the order of the phenotypic units. For example, the immunophenotype ‘CD4^+^FOXP3^+^CD25^hi^’ could be split into three phenotypic units (CD4^+^, FOXP3^+^ and CD25^hi^) and three smaller immunophenotypes, CD4^+^FOXP3^+^, FOXP3^+^CD25^hi^ and CD4^+^CD25^hi^, based on a two-layer combination of phenotypic units. Obviously, the functional states of immune cells corresponding to the child and parent entries were not identical. However, the immunophenotype split was important to understand the ‘3s’ model.

Therefore, each of the known immunophenotypes could be divided into one, two, three or more layers. Since the majority (~ 83%) of immunophenotypes were composed of no more than three genes (**Table S2**), we mainly analyzed them from layers 1-3, and more layers could be inferred from this. Therefore, we analyzed the impact of gene plasticity on the phenotypic composition.

The 1^st^ layer represented the involved marker genes themselves, as shown in **Table S2**. We examined their absolute GPL scores. The results showed that there were approximately 92.98% (159/171) for CD4^+^ T cells, 93.44% (114/122) for CD8^+^ T cells, 92.24% (107/116) for B cells, 80.77% (84/104) for NKs and 87.95% (73/83) for monocytes of the known marker genes with absolute GPL scores ≥ 50 in either array or RNA-Seq platforms (**Table S2**, Sheet 7). In addition, when the cutoff increased, there were still approximately 86.55% (148/171, CD4^+^ T cells), 87.70% (107/122, CD8^+^ T cells), 77.59% (90/116, B cells), 61.54% (64/104, NKs) and 79.52% (66/83, monocytes) of the known marker genes with an absolute GPL score ≥ 70 (**Table S2**, Sheet 7).

In T cells, there are many immunophenotypes containing neither CD4 nor CD8. Therefore, a total of 236 marker genes of these cells were also examined for GPLs in either CD4^+^ or CD8^+^ T cells, and we found that the marker genes with absolute GPL scores ≥ 50 accounted for approximately 92.80% (219/236) and 93.64% (221/236) of CD4^+^ and CD8^+^ T cells, respectively. When the cutoff increased, there were still approximately 84.32% (119/236, CD4^+^ T cells) and 85.59% (202/236, CD8^+^ T cells) of the marker genes with absolute GPL scores ≥ 70 in either cell type. Therefore, the results from all five cell types further confirmed that marker genes were generally highly plastic and that highly plastic genes were potentially suitable to label and classify immune cells.

A highly plastic gene implied that there should be at least two functional states corresponding to the high and low (or no) expression levels of the gene. Therefore, a combination composed of two highly plastic genes included four possible immunophenotypes, and a combination composed of three highly plastic genes included eight possible immunophenotypes, irrespective of the order of phenotypic units. For the two-layer combination, the known immunophenotypes were divided into 487 (CD4^+^ T cells), 241 (CD8^+^ T cells), 173 (B cells), 148 (NKs) and 26 (monocytes) nonredundant two-layer phenotypes, whereas for the three layers, the nonredundant combinational numbers were 809 (CD4^+^ T cells), 403 (CD8^+^ T cells), 148 (B cells) and 91 (NKs). For monocytes, there were few phenotypes containing multiple specific marker genes when the lineage markers CD14 and CD45 (also known as PTPRC/protein tyrosine phosphatase receptor type C) were removed (**Table S2**).

According to gene plasticity analysis, there were approximately 91.17% (CD4^+^ T cells), 94.19% (CD8^+^ T cells), 97.69% (B cells), 70.27% (NKs) and 76.92% (monocytes) of the two-layer phenotypes with both genes’ GPL scores simultaneously larger than or equal to 50 (**Table S2**, Sheet 7). For the three-layer phenotypes, the ratios were still remarkable, particularly for the cell types with a large number of marker genes, such as CD4^+^ T cells, CD8^+^ T cells and B cells. The ratios reached 91.72% (CD4^+^ T cells), 82.63% (CD8^+^ T cells) and 98.65% (B cells), and even when the threshold of GPL scores was set to 70, the supportive ratios were also notable, with 79.48% (CD4^+^ T cells), 59.55% (CD8^+^ T cells) and 51.35% (B cells) in the cells (**Table S2**, Sheet 7). There were relatively low numbers of marker genes, as well as the number of observed combinations, in NKs and monocytes. This may explain the slightly lower proportion of highly plastic gene combinations in these cells (**Table S2**, Sheet 7).

Therefore, the results suggest that continuous immunophenotypes could be defined and stratified by highly plastic genes. Marker genes of high plasticity formed a complex interaction network and constituted diversified phenotypes. This was consistent with the ‘3s’ model of this study. Because all the marker genes were derived from known functional subsets, the results suggest that the current model should be compatible with the traditional immune cell phenotypic classification and that novel phenotypes could be discovered *via* combinations of highly plastic genes.

The expressional highly plastic gene indicated that it had different expressional states at either the RNA or protein level and was able to subdivide the greater layers of phenotypes. Therefore, we used an experimental example to illustrate phenotypic subdivision. We selected three genes, CCR7 (C-C motif chemokine receptor 7), DPP4 (dipeptidyl peptidase 4, also known as CD26) and ITGA6 (integrin subunit alpha 6, also known as CD49f), in combination to define functional T-cell subsets, that is, CCR7/DPP4/ITGA6-based subsets. The absolute GPL scores of these genes were 93.7 (CCR7), 98.9 (DPP4) and 98.9 (ITGA6) in CD4^+^ T cells and 99.9 (CCR7), 98.8 (DPP4) and 98.8 (ITGA6) in CD8^+^ T cells according to RNA-Seq data (**Table S1**). Therefore, these genes were extremely highly plastic; in addition, their protein antibodies were easily obtained for flow cytometry assays.

**Figure 4:**
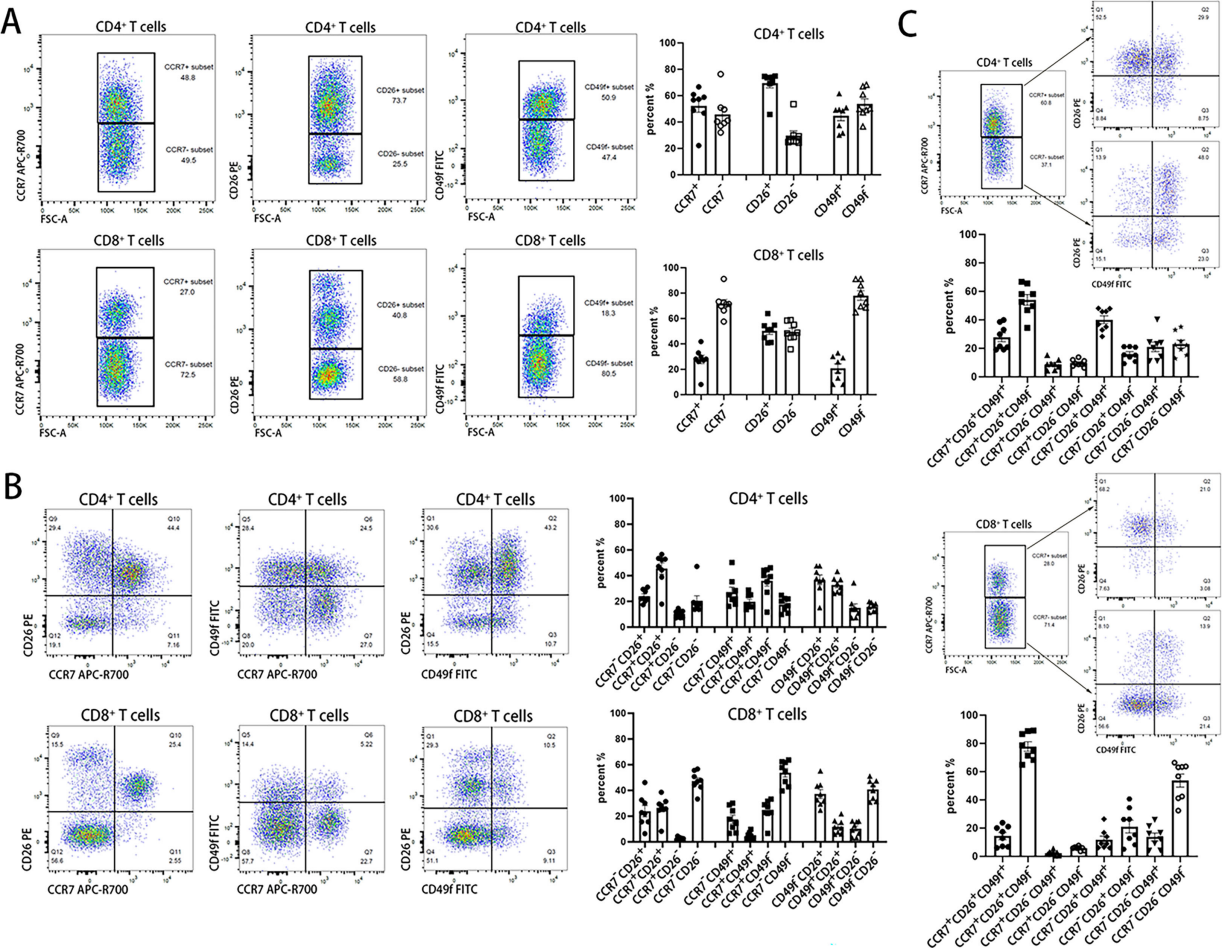
Three-layer classification of human T cells identified by CCR7, CD26 and CD49f. Three highly plastic molecules, CCR7, CD26, and CD49f are used to identify functional subsets in T cells. The 1^st^ layer in **(A)**, the 2^nd^ layer in **(B)** and the 3^rd^ layer in **(C)** use one, two and all three highly plastic molecules, respectively, to label the functional T-cell subsets.

As shown in **Figure 4A**, CCR7, CD26, and CD49f divided both CD4 and CD8 single-positive T cells into two subsets according to their positive and negative expression of a single marker gene. These subsets included CCR7^+^ and CCR7^-^ T cells, CD26^+^ and CD26^-^ T cells, and CD49f^+^ and CD49f^-^ T cells. There were more CD26^+^ but fewer CD49f^+^ cells in CD4^+^ T cells than in CD8^+^ T cells. CD26^+^ cells accounted for ~70% of CD4^+^ T cells, whereas CD49^-^ cells accounted for a similar proportion of CD8^+^ T cells. Interestingly, based on CD26 expression, T cells were divided into three functional subsets with high (CD26^hi^), moderate (CD26^int^) and low (CD26^lo^) expression, which was consistent with a previous report (35).

Next, a double combination of the three genes was used to confirm two-layer phenotypes. As shown in **Figure 4B**, a combination of any two genes could divide both CD4^+^ and CD8^+^ T cells into four subsets. For the three-layer subsets shown in **Figure 4C**, after CCR7 clustering of CD4^+^ T cells and CD8^+^ T cells, CD26 and CD49f were introduced to cluster both CCR7^+^ and CCR7^-^ T-cell subsets. This would produce a total of eight unique immunophenotypes in a three-gene combination when either of them was set as the 1^st^ layer. The most abundant cells were CCR7^+^CD26^+^CD49f^-^ in either CD4^+^ or CD8^+^ T cells, with an average proportion of 54% and 78% in CD4^+^ or CD8^+^ T cells, respectively.

Combined CD26 and CD49f also divided existing T-cell subsets into subdivisions. According to the expression of CD45RA and CCR7, human T cells can be divided into four subsets, including CD45RA^+^CCR7^+^ naïve (TN), CD45RA^-^CCR7^+^ central memory (TCM), CD45RA^-^CCR7^-^ effector memory (TEM), and CD45RA^+^CCR7^-^ effector memory reexpressing CD45RA (TEMRA) T cells (36). As shown in **Figure S3**, these cells could be further divided into more subdivisions based on the combined expression of CD26 and CD49f. In both CD4^+^ and CD8^+^ T naïve cells, CD49f^-^CD26^+^ cells had the highest cell proportion, reaching 80%. In CD4^+^ TCM and CD4^+^ TEM, CD49f^+^CD26^+^ has a relatively high cell proportion, approximately 40%-50%. In CD8^+^ TEM and CD8^+^ TEMRA cells, CD49f^-^CD26^-^ cells had the highest cell proportion, particularly with ~70% in CD8^+^ TEMRA cells. This result revealed that the additional highly plastic marker genes and known phenotypes could be further divided into subtle divisions, including potential novel phenotypes. Therefore, the characteristic of gene plasticity undoubtedly increases the diversity of immune cell phenotypes and the complexity of the immune system.

### Loss of phenotype accompanies the gain of phenotype

This classification system suggested that there was infinite diversity of immune cell phenotypes. The dynamic change in gene expression of a highly plastic gene was always accompanied by a set of correlated and anticorrelated genes, which resulted in cophenotypes and the loss of certain other phenotypes because of the loss of gene expression. An inevitable relationship in gene expression was previously called the ‘internal phenotype’ (22), which provided a new perspective of the rules for immune cell phenotypic conversion. Therefore, another distinctive feature of the current system was described as ‘loss of phenotype accompanying gain of phenotype’ or ‘phenotypic gain and loss’ in this study. The method of virtual sorting established in our previous studies provided a feasible and effective way to evaluate coexisting and lost phenotypes, which were represented by correlated and anticorrelated genes, respectively (22). All genes with absolute GPL scores greater than or equal to 50 in both array and HTS platforms were used for virtual sorting.

In total, we obtained 8,666 (CD4^+^ T cells), 10,676 (CD8^+^ T cells), 7,385 (B cells), 6,025 (NKs) and 10,107 (monocytes) genes with absolute GPL scores ≥50 shared by both platforms. These genes were subjected to virtual sorting as shown in **Figure S4**, and again we found that larger δ values were tightly associated with increased coexistence rates, whereas smaller δ values corresponded to higher mutually exclusive or APA rates (**Figure S5**), which was consistent with our previous results (21). Considering that the medians of the positive and negative δ values in all of the results were approximately 4 and -2, respectively, the δ values were set to be larger than or equal to 20 for the correlated genes and less than or equal to -10 for the anticorrelated genes in the current analysis. On the other hand, because negative marker genes were generally more difficult to find (20), if - 20 was set as the filter condition, the number of anticorrelated genes would be far less than the number of positively correlated genes. Under further strict filter conditions with Pearson correlation and cosine similarity (see Methods), there were finally 4,058 (CD4^+^ T cells), 3,895 (CD8^+^ T cells), 2,877 (B cells), 3,028 (NKs) and 4,183 (monocytes) genes with at least one correlated or anticorrelated gene (**Table S3**). For example, there were 99 genes, including 51 correlated and 48 anticorrelated genes, for ITGA6 in CD4^+^ T cells (**Table S3**).

Interestingly, there were many cytokine-encoding genes in the anticorrelated gene set, such as IL2, IL4, IL9, IL17A, IL21, IL22 and IFNG, suggesting that the loss of cytokine production should accompany the gain of high ITGA6 expression. ITGA6 was also observed in the anticorrelated genes of several cytokines, including IL2, IL3, IL9, IL10, IL13, IL21 and IL22 (**Table S3**), which suggests that high expression of these cytokines accompanies the loss of ITGA6 expression. Therefore, ITGA6 and these cytokines constituted mutually exclusive phenotypes. Similar to the function of ITGA6, which is a cell adhesion molecule, other molecules related to cell adhesion, such as EPHA4 (EPH receptor A4), AMIGO1 (adhesion molecule with Ig-like domain 1), DCHS1 (dachsous cadherin-related 1), EPHA1 and DSC1 (desmocollin 1), were significantly enriched in the correlated gene set of ITGA6 (**Table S3**). Therefore, the results suggest that there should be a mutually exclusive regulatory mechanism between cell adhesion and the inflammatory response mediated by cytokine secretion.

**Figure 5:**
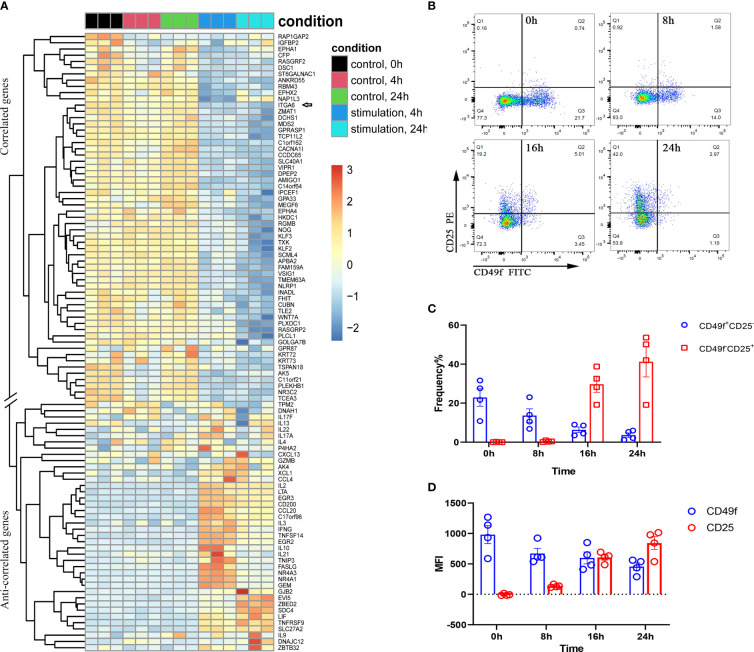
The correlated and anticorrelated genes of ITGA6 identified by virtual sorting are differentially expressed during T-cell activation. **(A)** Differential expression of the correlated and anticorrelated genes when naïve CD4^+^ T cells are activated by anti-CD3 and anti-CD28 at different time points. The dataset is derived from GSE39594. The arrow indicates the gene ‘ITGA6’. The correlated and anticorrelated genes with double slash separation are indicated on the left of the panel. **(B)** The scatter plot shows the decreased CD49f^+^ and increased CD25^+^ T cells after activation. **(C, D)** represent FACS results of anticorrelated expression between CD49f (ITGA6) and CD25 (IL2RA) in either positive cell frequency (proportion) **(C)** or MFI **(D)** in CD8^+^ T cells. In **(B–D)**, CD49f (ITGA6) is downregulated, whereas the anticorrelated gene CD25 is upregulated when CD8^+^ T cells are activated by PMA and ionomycin at different time points. CD8^+^ T cells were isolated from four independent healthy individuals.

ITGA6 mRNA showed relatively higher expression in naïve T cells based on the single-cell transcriptome (**Figure S6A**). We next analyzed the human naïve CD4^+^ T-cell activation transcriptome from the publicly available GEO dataset (ID: GSE39594). The normalized expression values from the data submitter were directly used for analysis. As shown in **Figure 5A**, when naïve CD4^+^ T cells were activated by anti-CD3 and anti-CD28, the genes correlated with ITGA6 tended to be downregulated upon stimulation, whereas the anticorrelated genes showed an overall upregulation. In addition, the single-cell transcriptome in human T cells also revealed similar expression patterns among the correlated genes and anticorrelated genes (**Figure S6A**). In CD8^+^ T cells, under less strict conditions, many cytokine-encoding genes, such as CSF1 (colony stimulating factor 1), IFNG, IL13, LIF (leukemia inhibitory factor), TNF, LTA (lymphotoxin alpha) and CCL22, were also observed to be anticorrelated to ITGA6. We found that the cell surface molecule IL2RA (also called CD25) was one of the topmost anticorrelated genes of ITGA6 in CD8^+^ T cells. The anticorrelated expression of IL2RA and ITGA6 was further confirmed at the protein level (**Figures 5B–D**). After stimulation with PMA and ionomycin, the frequency (proportion) of ITGA6^+^ T cells, as well as the expressional intensity of ITGA6 as indicated by the mean fluorescent intensity (MFI), gradually decreased after 0, 8, 16 and 24 hours of stimulation; however, as expected, IL2RA increased gradually in either the expressional proportion or intensity in response to stimulation (**Figures 5B–D**). This result further confirmed that the acquisition of ITGA6 expression was accompanied by an inevitable loss of IL2RA expression at either the mRNA or protein level.

**Figure 6:**
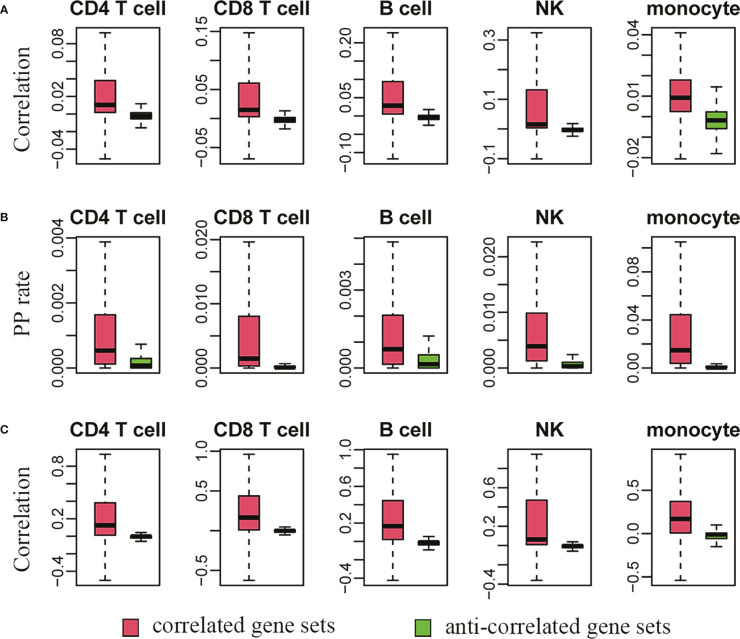
The single-cell transcriptome supports the virtual sorting results derived from bulk RNA sequencing data. **(A, C)** indicate the distributions of Pearson correlation coefficients before and after imputation by SAVER. A higher correlation **(A)** and coexistence rate or present-present/PP rate **(B)** between highly plastic genes and their correlated genes are also observed in the single-cell dataset. After imputation, the correlation strength is further greatly improved **(C)**.

Next, we systematically evaluated the results in **Table S1** based on the single-cell transcriptome of human PBMCs. Because the results in **Table S1** were from bulk RNA-Seq, an evaluation based on single cells represented an independent way to check the reliability. Two methods, including Pearson correlation and present-present (PP) rate (or coexistence rate), were used (24). For each gene pair between the highly plastic genes and the correlated or anticorrelated genes, the former represented linear coexpression, while the latter indicated a cell proportion with coexistence of the genes based on their unique molecular identifier (UMI) counts simultaneously larger than zero in the current situation. As shown in **Figure 6**, there were remarkable differences in the distributions of either Pearson correlation coefficients (**Figure 6A**) or PP rates (**Figure 6B**), indicating stronger correlation and coexistence in the correlated gene sets than in the anticorrelated gene sets in each cell type. However, the absolute values of either the correlation coefficients or the PP rates were small, which was caused by the generally large proportion of zero values or dropouts in single-cell data owing to the low initial amounts of RNA obtained from a single cell (37).

Therefore, we further used SAVER (single-cell analysis *via* expression recovery), an expression recovery method in noisy and sparse single-cell RNA-seq data (38), to recover the true gene expression in each cell type. After imputation by SAVER, the correlation strength was greatly improved for the correlated gene sets, but there was no obvious improvement in terms of Pearson correlation for the anticorrelated gene sets (**Figure 6C**). This was exactly in line with the current logic and our expectation because the anti-correlation identified by virtual sorting represented typical nonlinear relationships (21, 22), and the results of anticorrelation were filtered by cosine similarity, which was not a measure of linear association such as Pearson correlation. Therefore, the above results from either single gene or systematic analysis of thousands of highly plastic genes supported the gain and loss of phenotypes accompanying the dynamic expressional change of highly plastic genes.

### Single-cell transcriptome combined with gene plasticity analysis facilitates the discovery of novel functional subsets of immune cells

The current phenotypic classification system is an open system. In addition to the ability to explain phenotypic diversity, indicate subdivisions and imply phenotypic regulation during cell state conversion, the system functioned as a discovery model and was able to predict and identify novel immune cell subsets. Highly plastic genes were described to be suitable for marker genes to label immune cell subsets in our previous study (21). The next analysis focused on single-cell omics, which greatly assisted in discovering novel immune cell subsets based on gene plasticity analysis.

It is logically reasonable and feasible to use highly plastic genes, particularly extremely plastic genes with expressional bipolar states, to classify immune cells and predict novel functional subsets. However, during routine isolation of target cells from blood or other tissues by flow cytometry, the purity of target cells is usually difficult to reach 100%. Therefore, cell samples with insufficient cell purity during bulk RNA-Seq or microarray assays may increase gene plasticity, at least for some genes that are highly expressed in unwanted or contaminated cells. Cell purity has a minor influence when the genes are expressed at low levels in the target cells because they also show low expression in the observed bulk samples; however, cell purity must be considered when high expression states are observed in bulk samples, which might be caused by other cell contamination. Single-cell RNA sequencing (scRNA-Seq) examines gene expression at single-cell resolution and has advantages for cell type-specific analysis, particularly for genes with high expression states. Therefore, we used single-cell data to trace the highly plastic states with polarized gene expression (or polarized genes for short) and to discover novel functional immune cell subsets.

First, among the highly plastic genes with absolute GPL scores ≥50 shared by both array and HTS platforms, we further set a strict condition with the requirement of the maximum rank score ≥90 in both platforms to screen genes of interest. This meant that these polarized genes had the potential to be dramatically expressed and belonged to the top 10% of genes with the highest expression under certain conditions. As a result, 2,600 (CD4^+^ T cells), 2,871 (CD8^+^ T cells), 2,209 (B cells), 1,268 (NKs) and 3,110 (monocytes) genes were identified to have polarized expression supported by both platforms (**Table S4**).

Next, a total of 17 single-cell datasets related to human PBMCs were first collected, which corresponded to Dataset 1 to Dataset 17 (**Table S4**, sheet 6). These datasets comprised a total of 2,563,048 single cells, including 909,248 CD4^+^ T cells, 557,317 CD8^+^ T cells, 482,179 B cells, 145,623 NK cells and 468,717 monocytes (**Table S4**). However, these single cells from PBMCs only represented gene expression in the blood tissue and could not cover extensive functional states. Therefore, we further collected an additional 21 single-cell datasets derived from other tissues, including several tumors, such as prostate cancer, hepatocellular carcinoma, lung cancer, gastric cancer, breast cancer, melanoma, basal cell carcinoma, squamous cell carcinoma and colorectal cancer. These additional datasets comprised a total of 385,832 single cells, including 12,2013 CD4^+^ T cells, 114,021 CD8^+^ T cells, 93,542 B cells, 42,627 NK cells and 13,629 monocytes (**Table S4**). We confirmed that there was a generally close positive correlation between the expression proportion and the average expression intensity at the whole gene level in each cell type. Therefore, the percentage of gene expression could be used to evaluate to what extent genes were expressed.

As shown in **Table S4**, the genes showed variable expression in different datasets, suggesting that gene plasticity states should be closely associated with conditions. A high proportion of expression in single cells indicated a high plasticity state. For example, under a filter condition of 25% single cells with detectable expression, 797 (CD4^+^ T cells), 887 (CD8^+^ T cells), 664 (B cells), 408 (NKs) and 1,212 (monocytes) genes were identified to be highly expressed in at least one dataset (**Table S4**). Undoubtedly, the lower the filtering conditions were, or the greater the single-cell datasets were collected for the analysis, the better was it supported in the numbers of the high plasticity genes in **Table S4** by single cells. The following represent several examples of novel functional subsets supported by the single-cell transcriptome.

**Figure 7:**
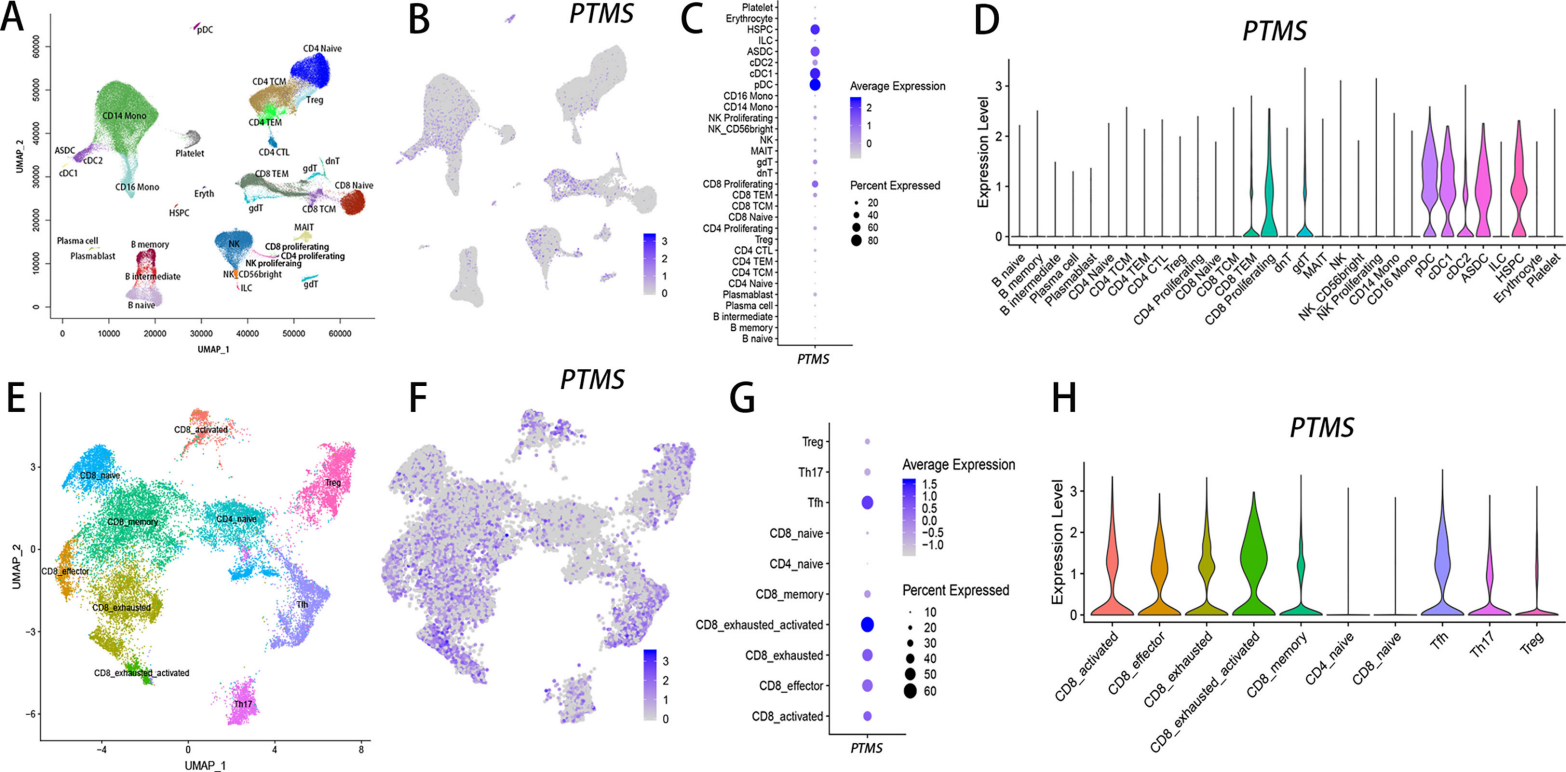
PTMS-positive T-cell subsets are enriched in squamous cell carcinoma (SCC) based on single-cell transcriptomic analysis. UMAP plots were used to show immune cell subsets in the datasets from multimodal human PBMCs **(A)** and SCC **(E)**. The expression levels of PTMS are illustrated by feature plots **(B, F)**, dot plots **(C, G)** and violin plots **(D, H)** in either PBMCs **(B–D)** or SCC **(F–H)**.

Parathymosin (PTMS) is a small acidic nuclear protein. Although it was originally isolated from the rat thymus, PTMS was not expressed in thymocytes in previous reports (39, 40). However, PTMS was a highly plastic gene in T cells (**Tables S1** & **S4**), which meant it was not widely expressed under all kinds of conditions, as was done for lowly plastic genes. The highly plastic characteristic of PTMS implied that it should be highly expressed under certain conditions but show very low or even no expression under some other conditions. Based on the multimodal single-cell dataset (Dataset 1 in **Table S4**), which comprises over 161,000 single cells from PBMCs (41), PTMS was expressed at low levels in B cells, T cells, monocytes and NKs but showed the highest expression in proliferating CD8^+^ T cells (**Figures 7A–D**). Interestingly, PTMS was also highly expressed in hematopoietic stem and progenitor cells (HSPCs) and dendritic cells (DCs), including plasmacytoid dendritic cells (pDCs) and conventional DCs (cDCs), as well as ASDCs, which are defined by the expression of AXL (AXL receptor tyrosine kinase) and SIGLEC6 (sialic acid binding Ig-like lectin 6) (42). However, when tracing the highly plastic state of PTMS using tumor single cells, we found that PTMS was highly expressed in tumor-infiltrating lymphocytes (TILs), particularly TILs from squamous cell carcinoma (SCC), such as Tfh, Th17, Treg, and activated and exhausted CD8^+^ T cells (**Figures 7E–H**), whereas PTMS showed relatively low expression in naïve T cells (**Figures 7G, H**).

We examined what phenotypes were lost (i.e., missing phenotypes) and gained (i.e., cophenotypes) during the acquisition of high levels of PTMS expression. Under the strict filter conditions mentioned above, there were no genes with an expressional positive correlation to PTMS in T cells (**Table S3**). However, when we relaxed the filtering conditions and set the δ value ≥10 for the correlated gene set during virtual sorting, 166 and 147 genes were identified in the CD4^+^ and CD8^+^ single-positive T cells, respectively (**Table S5**), and 66 genes were shared by both cell types. Functional enrichment analysis revealed that biological processes related to the cell cycle, such as cell division, mitotic cytokinesis and chromosome segregation, were significantly enriched in the correlated gene sets in both cell types (**Table S5**). For the anticorrelated genes of PTMS, genes related to the inflammatory response, such as S100A8 (S100 calcium binding protein A8) and S100A9, were downregulated in PTMS^+^ T cells (**Tables S3** & **S5**). Functional enrichment further confirmed that biological processes, such as neutrophil aggregation, neutrophil chemotaxis and inflammatory response, were significantly enriched in the anticorrelated genes in CD4^+^ T cells (**Table S5**). This suggests that PTMS are highly plastic cell cycle–associated molecules (HPCCMs) based on our previous definition (22) and that PTMS^+^ T cells should be closely associated with a proliferative phenotype.

Although the functional understanding is limited, studies have shown that PTMS is involved in the replication of active chromatin (43), in the remodeling of higher-order chromatin structure *via* interaction with histone H1 (44) and in the regulation of inflammation *via* inhibition of the transcriptional activity of NF-κB (45). These studies support our above results regarding functional clues. Moreover, in a recent study, PTMS was found to be a brain-secretory protein with a neuroprotective role; neurons in various brain regions not only released PTMS but also received PTMS (46). Currently, there are no reports about the functions of PTMS in the immune system; therefore, the exact role of PTMS^+^ T cells in tumor immunity still awaits further investigation.

**Figure 8:**
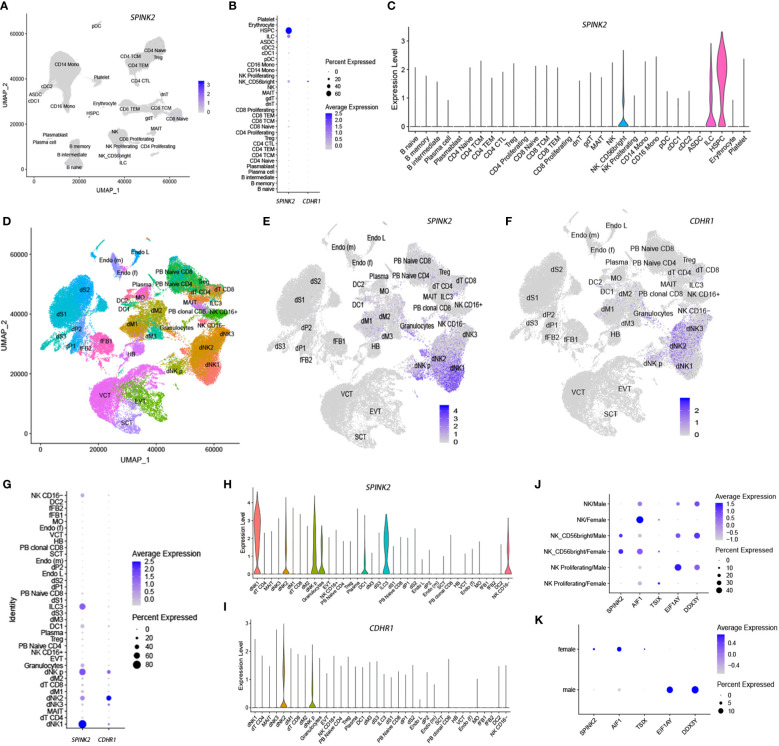
SPINK2 and CDHR1 single-positive NK cells are enriched in the decidua based on single-cell transcriptomic analysis. The indicated gene expression levels are illustrated by feature plots **(A, E, F)**, dot plots **(B, G)** and violin plots **(C, H, I)** in both PBMCs **(A–C)** and decidua **(E–I)**. The UMAP plot in **(D)** shows cell types in the decidua, as well as in peripheral blood (PB) for some cell types. SPINK2 and the correlated gene AIF1 show higher expression in female NKs, as supported by either PBMC **(J)** or lung cancer **(K)** datasets. The genes TSIX, EIF1AY and DDX3Y represent positive controls to indicate female (TSIX) and male (EIF1AY, DDX3Y) NKs. Some cell abbreviations are shown as follows. dNK1-3, decidual NK-cell cluster 1-3; dT CD4, decidual CD4^+^ T cells; MAIT, mucosal-associated invariant T cells; dM1-3, decidual macrophage cluster 1-3; dNK p, decidual proliferative NK cells; EVT, extravillous trophoblast; DC, dendritic cells; dS1-3, decidual stromal cell cluster 1-3; dP1-2, decidual perivascular cell cluster 1-2; Endo L, lymphatic endothelial cells; Endo (m), maternal endothelial cells; SCT, syncytiotrophoblast; HB, Hofbauer cells; VCT, villous cytotrophoblast; Endo (f), fetal endothelial cells; MO, maternal macrophages; fFB1-2, fetal fibroblast cluster 1-2.

The serine peptidase inhibitor Kazal type 2 (SPINK2) and cadherin-related family member 1 (CDHR1) were highly plastic in NKs (**Tables S1** & **S4**). Although both genes showed low expression in NKs from PBMCs (**Figures 8A–C**), they were highly expressed in decidual natural killer cells (dNKs) (**Figures 8E–I**) based on a recent dataset related to the maternal-fetal interface in humans (47). Interestingly, SPINK2 and CDHR1 were preferentially expressed in different dNK subsets, that is, dNK1 and dNK2, respectively. SPINK2 showed higher expression in proliferating dNKs (dNK p) than CDHR1 (**Figures 8G–I**). Further analysis of gain and loss of phenotypes revealed that SPINK2^+^ NKs and CDHR1^+^ NKs showed distinct functional characteristics. The correlated genes of SPINK2 in NKs were significantly enriched in biological processes, including cell division, mitosis, cell cycle and chromosome segregation (**Table S5**), whereas these processes were significantly enriched in the anticorrelated genes of CDHR1 (**Table S5**), suggesting diversified phenotypes between SPINK2 and CDHR1 single-positive dNKs.

SPINK2 is a secretory protein and a strong inhibitor of acrosin-trypsin. SPINK2 deficiency causes male infertility, suggesting an important role in male reproduction (48, 49). SPINK2 is highly expressed in the testis based on several public databases, such as GTEx (50), but it was also highly expressed in maternal dNKs because of gene plasticity, particularly in dNK1 cells. The dNK1 subset is suggested to play a role in recognizing and responding to placental extravillous trophoblast cells (EVTs) (47). Interestingly, we found that there were several genes, including *EIF1AY* (eukaryotic translation initiation factor 1A Y-linked), *ZFY* (zinc finger protein Y-linked), *KDM5D* (lysine demethylase 5D), *USP9Y* (ubiquitin specific peptidase 9 Y-linked), *RPS4Y1* (ribosomal protein S4 Y-linked 1) and *DDX3Y* (DEAD-box helicase 3 Y-linked), which are located on chromosome Y in the anticorrelated gene set of SPINK2 (**Table S5**). Considering that female-specific genes on chromosome X are generally anticorrelated to male-specific genes on chromosome Y during virtual sorting (22), we further examined whether *SPINK2*, which is located on chromosome 4, showed sex-associated expression in NKs. Therefore, we selected two datasets from peripheral blood (Dataset 1) and lung cancer (Dataset 20), which comprised enough NKs (**Table S4**). The former included NKs from 6 males and 2 females, while the latter included NKs from 38 males and 18 females. As shown in **Figure 8**, SPINK2 and at least one of the correlated genes, AIF1 (allograft inflammatory factor 1), showed higher expression in female NKs than in male NKs from either PBMCs (**Figure 8J**) or the microenvironment of lung cancer (**Figure 8K**). This result suggests that sex-associated regulation and SPINK2^+^ NKs play an important role in female reproduction.

In contrast to SPINK2, the correlated genes of CDHR1 were enriched in biological processes, such as the innate immune response, defense response, leukocyte migration involved in the inflammatory response, complement activation and cell-cell adhesion (**Table S5**). Therefore, CDHR1 is a highly plastic immune and defense response-associated molecule (HPIDM) based on our previous definition (22). CDHR1 is a member of the cadherin superfamily and is responsible for calcium-dependent cell-to-cell adhesion, which serves an array of essential roles, including structural aggregation, cell migration, cell-cell signaling and cell polarity. CDHR1 showed high expression in several human tissues, such as skin, gut and brain, based on the GTEx database. It is also expressed in photoreceptors and ganglion cells in retinal tissue, and its function is mainly focused on retinal cadherinopathies, such as cone-rod dystrophy, late-onset macular dystrophy, and retinitis pigmentosa (RP) (51).

As a cell surface molecule, the specific high expression of CDHR1 in dNKs reminded us of CD49a (also called ITGA1), an integrin alpha subunit that binds collagen and laminin, which is considered a marker of tissue-resident NK (trNK) cell subsets (52). In the human uterus, there are a large number of CD49a^+^ trNK cells, which are involved in fetal development (53). However, based on the current analysis, as a highly plastic molecule, CD49a showed wide expression in multiple tissues, such as gastric cancer, basal cell carcinoma and colorectal cancer, whereas high expression of CDHR1 was restricted to decidual tissue (**Table S4**, **Figure S6B**), suggesting its specific functional significance in the decidua. Therefore, the current study suggests the diversified roles of SPINK2^+^ and CDHR1^+^ single-positive dNKs and their important role in maternal-fetal interactions.

For B cells, an example of DCAF12 (DDB1 and CUL4-associated factor 12)-positive B cells is shown in **Figure S7**. DCAF12 was highly expressed in the B cells of gut tissue (Dataset 30) (**Table S4**). DCAF12 is a cofactor of cullin-4 (CUL4) ubiquitin ligase complexes and mediates substrate recognition and recruitment. Known substrates of DCAF12 include melanoma antigen gene (MAGE) family members (54) and Moloney leukemia virus 10 (MOV10) (55). DCAF12 was initially identified as a regulator of tissue growth and apoptosis in Drosophila melanogaster (56). A recent study from *Dcaf12* knockout (KO) mice revealed that *Dcaf12* deficiency led to a decreased sperm count, dysregulation of immune cell populations, and increased splenocyte apoptosis after T-cell activation (55).

DCAF12 is highly plastic in immune cells (**Table S1**). Although DCAF12 showed low expression in peripheral B cells (**Figures S7A–C**), it truly showed high expression levels in cycling immune cells, such as cycling plasma cells, myeloid cells and B cells (**Figures S7D–G**), suggesting a functional association with cell proliferation. The association between proliferative phenotypes and high expression of DCAF12 in B cells was also implied based on the correlated gene analysis, which revealed that cell cycle-relevant biological processes were significantly enriched in the correlated gene set of DCAF12 (**Table S5**). In addition, the correlated and anticorrelated genes showed compatible and exclusive expression patterns, respectively (**Figure S7H**), suggesting the reliability of functional evaluation by virtual sorting.

None of these subsets have been studied in the field. Therefore, a global view of gene plasticity provides a feasible and efficient way to identify novel functional subsets of immune cells. In addition, omics big data, particularly single-cell omics, provide an opportunity to trace and validate where these subsets may exist.

## Discussion

It is worth considering why there are so abundant functional subsets of immune cells. In the current study, we proposed a novel framework for immune cell classification based on gene plasticity. It reflects the different phenotypes or functional cell states under a series of various conditions. The system provides a reasonable explanation of phenotypic diversity and plasticity. In this system, we paid less attention to the cell type hierarchy as the Cell Ontology does. Cell Ontology represents an annotation system of known cell types based on their properties, such as the main functions and histological and developmental lineage classes; the ontology system is organized as a directed acyclic graph (57). In the era of massive single-cell omics data, cell ontologies, a structured controlled vocabulary for cell types in animals, provide a powerful way to understand such knowledge about the cellular composition of the human body (58), which in turn facilitates cell type annotation during single-cell omics data analysis. However, Cell Ontology is not a system aimed at novel subset discovery and provides a limited explanation of cell states (58). It is unable to explain the diversity of immune cell phenotypes and incapable of analyzing cophenotypes and the loss of phenotypes. Therefore, it is different from the purpose and application of the current system. However, the phenotypes identified through the current system can finally enter Cell Ontology once the required knowledge has been addressed. In this regard, the two systems work synergistically.

One of the two core points of the system is that small subsets can be divided from the greater subset based on their highly plastic genes. The divisions are mainly based on systematic analysis rather than referring to infinite immune cell phenotypes in only one individual or under extremely limited conditions. The combinations among highly plastic genes constitute rich and diverse phenotypes. Gene plasticity represents a series of continuous plastic states, such as high, low and intermediate gene plasticity states. During the change among the states, such as from high to low expressional levels, or *vice versa*, correlated and anticorrelated genes change in different directions. During this process, the inevitable intergenic relationship among genes implies an inherent regulatory mechanism, which we call internal phenotype regulation (22). Therefore, in addition to the diversity, layerability and predictability inferred by the system, it reflects the underlying regulation of immune cell phenotypes. In addition, it is conceptually simple and easy to connect with the known concept of cell plasticity. The model is also suitable for large-scale systematic computer analysis and prediction.

Based on gene plasticity, the cell functional state under a specific condition could be represented by a group of highly plastic genes accompanied by a group of correlated (cophenotypic) and anticorrelated (mutually exclusive phenotypic) genes. These highly plastic genes are in their respective plastic states with either high, moderate or low levels, representing a result of multiple intergenic interactions and compromise. However, for the observer, due to the limited experimental conditions, the observed cell states at the population level are limited. Similarly, we could not observe all of the correlated and anticorrelated genes simultaneously under limited conditions. However, low-plasticity genes are generally either highly expressed or expressed at low levels in a cell type (21). Therefore, the lowly plastic but highly expressed genes should also play an important role in functional maintenance across all cell states.

Although we analyzed only the five main cell types, including human CD4^+^ T cells, CD8^+^ T cells, NKs, B cells and monocytes, the gene plasticity model, which represents a general rule in cells, is also suitable for other immune cells, such as macrophages and DCs, and even for nonimmune cells, such as fibroblasts and even cancer cells. It is an open system and compatible with the traditional immune cell category. This is because the traditional lineage marker genes are lowly plastic in the corresponding cell types (21) and are also highly plastic if they are placed in a higher layer. This suggests that gene variability analysis could be carried out at different layers, and gene plasticity would change accordingly. For example, the lineage marker CD3D is lowly plastic in T cells but shows increased plasticity in cells mixed with T cells and non-T cells, to some extent representing different proportions of CD3D^+^ T cells. Similarly, when we mention a high plasticity gene, the proportions of the cells with positive expression of the gene in a large cell population will also be different under various conditions. Therefore, gene plasticity is always associated with layering and the accompanying plasticity transition. Correlation analysis with stratification may help to more accurately analyze cophenotypes and mutually exclusive phenotypes. Moreover, this model does not particularly emphasize which gene acts as the starting layer. However, under specific conditions, the gene order based on the number of cells is worth considering.

Therefore, when classifying traditional immune cell subsets, such as naïve, memory and effector T cells, it is necessary to identify the high plasticity genes in these cells. Massive omic big data provide an effective way for the existence of highly plastic genes. However, whether there are enough highly plastic genes depends on the experimental conditions and the amount of detected data. Although the traditional classification model can well reflect the lineage relationship between different immune cell types, it cannot well explain the high heterogeneity and plasticity of immune cells. Plasticity implies dynamic characteristics, stratification and subdivision as well as convertibility, whereas heterogeneity implies dissimilarity and diversity. The dissimilar phenotypes in the same or different layers represent heterogeneity. Therefore, plasticity explains the underlying mechanism of heterogeneity.

However, the current gene plasticity based on rank profiles may also have limitations. In this study, we focused on genes with high plasticity. However, when the change in expression level has a weak impact on the rank change, gene plasticity analysis may have limited usage for discovering novel immune cell subsets and phenotypes. For example, when the expression level of an extremely highly expressed gene is further increased, the rank score may not be accompanied by a large change or may even remain the same. On the other hand, gene plasticity analysis is subject to the experimental conditions associated with samples. For example, when the highly or lowly expressed states of a certain gene remain unidentified or the related samples have not been collected in the current analysis, this may affect the observation of highly plastic states of the gene. In this study, although each cell type has hundreds of samples derived from different conditions, it may still affect the observation of some highly plastic genes.

Although the current model proposes infinite immune cell phenotypes, in practice, the discovery of immune cell subsets remains limited due to technical limitations. In a recent report, major T-cell subsets and immunophenotypes of human peripheral blood and lymph nodes were detected using 31-parameter (29-color) flow cytometry (59). However, the number of highly plastic genes is much greater than the fluorescent colors currently used in flow cytometry. With the development of flow technology, there will be more technical methods to jointly detect more parameters and fluorescence in the future. In addition, the availability of applicable monoclonal antibodies is another problem because in the current model, it is not limited to secreted proteins, cell membrane molecules and transcription factors. There are very limited monoclonal antibodies labeled by fluorescein for intracellular molecules on the market.

In recent years, single-cell sequencing technology has been widely used in the discovery of novel immune cell subpopulations. Because mixed cell types can be clearly identified in single-cell technologies, it could be better to carry out gene plasticity analysis in a single-cell transcriptome than in bulk RNA-Seq. However, due to current technical limitations, the number of genes detected per cell is extremely limited, ranging from tens to thousands. In many studies, cells with a detection number of over 5,000 genes are often considered doublet or multiplet cells and are removed in quality control, while cells with less than 200 detected genes are also removed. Genes detected by single-cell sequencing are often highly expressed genes but still retain missing values (dropouts). Different cell types or their subsets exist in the form of cell clusters, which are complex to calculate and closely related to sample batches, the number of genes tested, algorithms, and so on (37, 60). Therefore, a new cluster does not necessarily represent a novel subset during single-cell analysis. In single-cell research, the higher the level of gene expression is, the greater the variation in gene expression (61). This is different from the generally low plasticity of highly expressed genes in gene plasticity analysis. Single-cell technologies have limited resolution in the definition of immune cell subsets because cell clustering is performed at the population level rather than at the single gene level. In addition, single-cell technologies cannot effectively distinguish cell subsets for highly homogeneous cell structures. However, we are convinced that single-cell technologies truly provide an effective way to validate the current gene plasticity model.

Gene plasticity emphasizes a dynamic but not static process. Corresponding to multiomics, gene plasticity can be reflected at multiple levels, such as expressional gene plasticity at the mRNA and protein levels and epigenetic gene plasticity at the methylation level in our recent study (62). All plastic genes constitute the plasticitome. Therefore, a systematic analysis of gene plasticity will directly contribute to the development of ‘plasticitomics’, which focuses on the study of gene plasticity at multiple levels to explore the dynamic change rules of life molecules and the underlying mechanisms, as well as applications based on gene plasticity. Currently, massive amounts of omics big data provide an opportunity for plasticitomics research. The integration of multiomics data for the classification of the current model will also be an exploration direction.

Similarly, the correlated genes identified in this study contribute to cophenotypes and constitute the inclusive omics, which means that these genes are compatible with each other and can be highly expressed simultaneously in a cell or expressed as ‘high level compatibility’. For the genes with expressional compatibility, it does not mean that these genes are simultaneously highly expressed under any conditions. However, genes with mutually exclusive expression constitute the exclusive omics, which means that these genes cannot be simultaneously expressed at high levels or expressed as ‘high level exclusion’. Therefore, for mutually exclusive gene pairs, only one gene is highly expressed, but the other shows low or no expression. Both the inclusive- and exclusive-omes belong to the plasticitome and can exist at low levels or be expressed as ‘low level coexistence’. Therefore, similar to gene plasticity, inclusiveness and exclusiveness can be expressed at multiple levels, such as the mRNA, protein and epigenetic levels. When a global view of a series of cell states in terms of gene expression, methylation and other events is used, gene plasticity analysis provides a novel perspective to classify the functional states of both immune cells and nonimmune cells.

## Data availability statement

The original contributions presented in the study are included in the article/**Supplementary Material**. Further inquiries can be directed to the corresponding author.

## Ethics statement

The studies involving human participants were reviewed and approved by Peking University Biomedical Ethics Committee. The patients/participants provided their written informed consent to participate in this study.

## Author contributions

PW designed the study and wrote the manuscript. YH did the verification experiments and performed data analysis under the help of PW. CL and WH provided help and suggestion during the study and manuscript preparation. Conceptualization, funding acquisition and project administration was from PW. All authors contributed to the article and approved the submitted version.
