# Corrigendum: A theoretical framework of immune cell phenotypic classification and discovery

**DOI:** 10.3389/fimmu.2023.1234508

**Published:** 2023-06-13

**Authors:** Yuzhe Hu, Chen Liu, Wenling Han, Pingzhang Wang

**Affiliations:** ^1^ Department of Immunology, NHC Key Laboratory of Medical Immunology (Peking University), School of Basic Medical Sciences, Peking University Health Science Center, Beijing, China; ^2^ Peking University Center for Human Disease Genomics, Beijing, China; ^3^ Department of Clinical Laboratory, Peking University People’s Hospital, Beijing, China

**Keywords:** gene plasticity, plasticity-based classification, plasticitome, plasticitomics, immunophenotype, parathymosin, SPINK2, CDHR1

In the published article, there was an error in the **Introduction**, paragraph 4 was inserted in error. The following sentence should be removed:

“The sequence of symbols is not consistent with the original manuscript. It should be as follows.”

The authors apologize for this error and state that this does not change the scientific conclusions of the article in any way. The original article has been updated.

