# Supplementary material for: A theoretical framework of immune cell phenotypic classification and discovery
Source: Front Immunol. 2023 Mar 2;14:1128423. doi: 10.3389/fimmu.2023.1128423 (PMC10018129; doi:10.3389/fimmu.2023.1128423)
Supplement: Supplementary file 1 [file DataSheet_1.docx]

**Supplementary Figures and legends**

Figure S1. Scatter plots show the distributions of ARSs calculated using the common genes in RNA-Seq and microarray datasets.

Figure S2. Examples of extremely plastic genes as markers of known CD4^+^ T cell subsets.

Figure S3. T-cell subset subdivisions identified by CD26 and CD49f.

Figure S4. A schematic diagram showing the virtual sorting process used in this study.

Figure S5. The box plot shows the relationship between Delta and coexistence or mutually exclusive rate.

Figure S6. The dot plot shows the expression profiles of the indicated genes in different single-cell datasets.

Figure S7. DCAF12-positive B cells are enriched in the gut based on single-cell transcriptomic analysis.

**1. Figure S1**

**
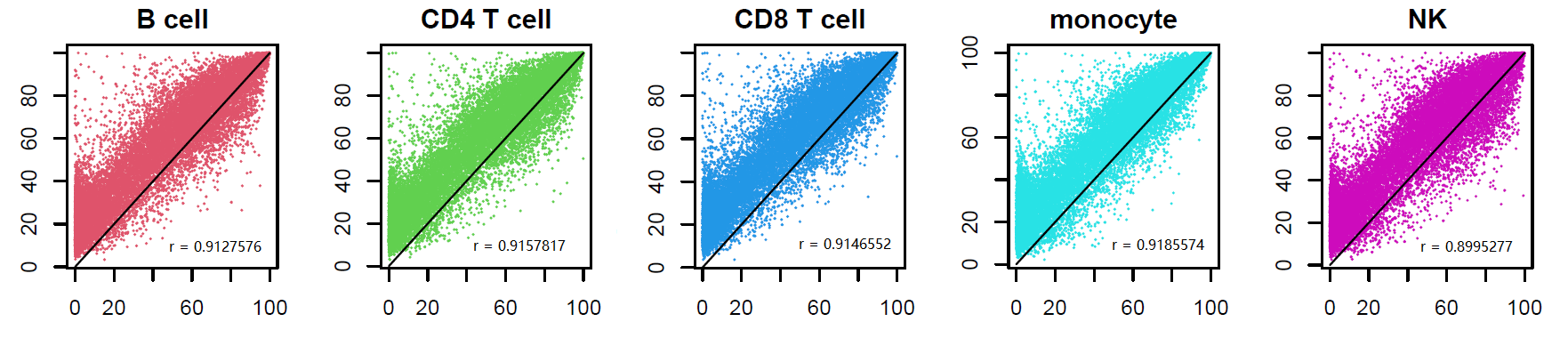
**

**Figure S1. Scatter plots show the distributions of ARSs calculated using the common genes in RNA-Seq and microarray datasets.** A total of 16,513 common genes were extracted and their average rank scores (ARSs) were re-calculated. In each panel, the *x*-axis indicates ARSs from RNA-Seq data, while the *y*-axis indicates ARSs from microarray data. Each point represents an ARS value. The Pearson coefficient (r) is shown in each panel.

**2. Figure S2**

**
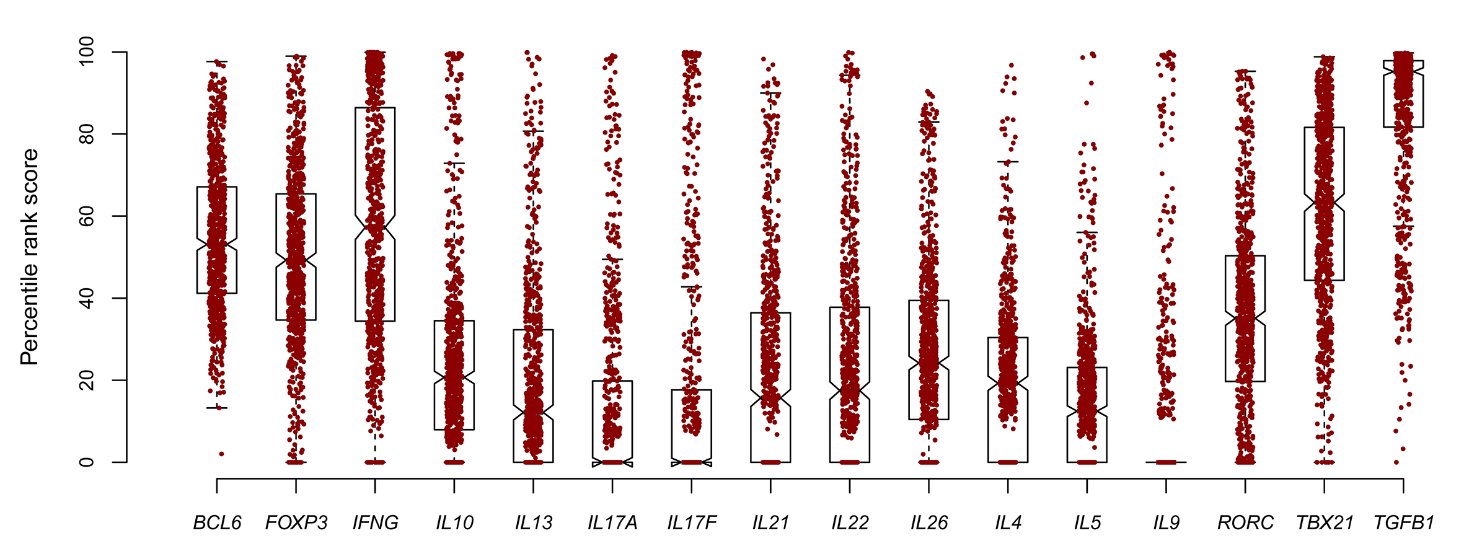
**

**Figure S2.** **Examples of extremely plastic genes as markers of known CD4+ T cell subsets.** Box plots are used to show the rank score distribution of selected known marker molecules with extremely high plasticity in CD4^+^ T cells. The bottom and top of the boxes represent the first and third quartiles, and the band inside each box represents the median (2^nd^ quartile) of rank scores across samples. The figure provides a global view of the continuous gene plasticity states of the selected genes.

**3. Figure S3**

**
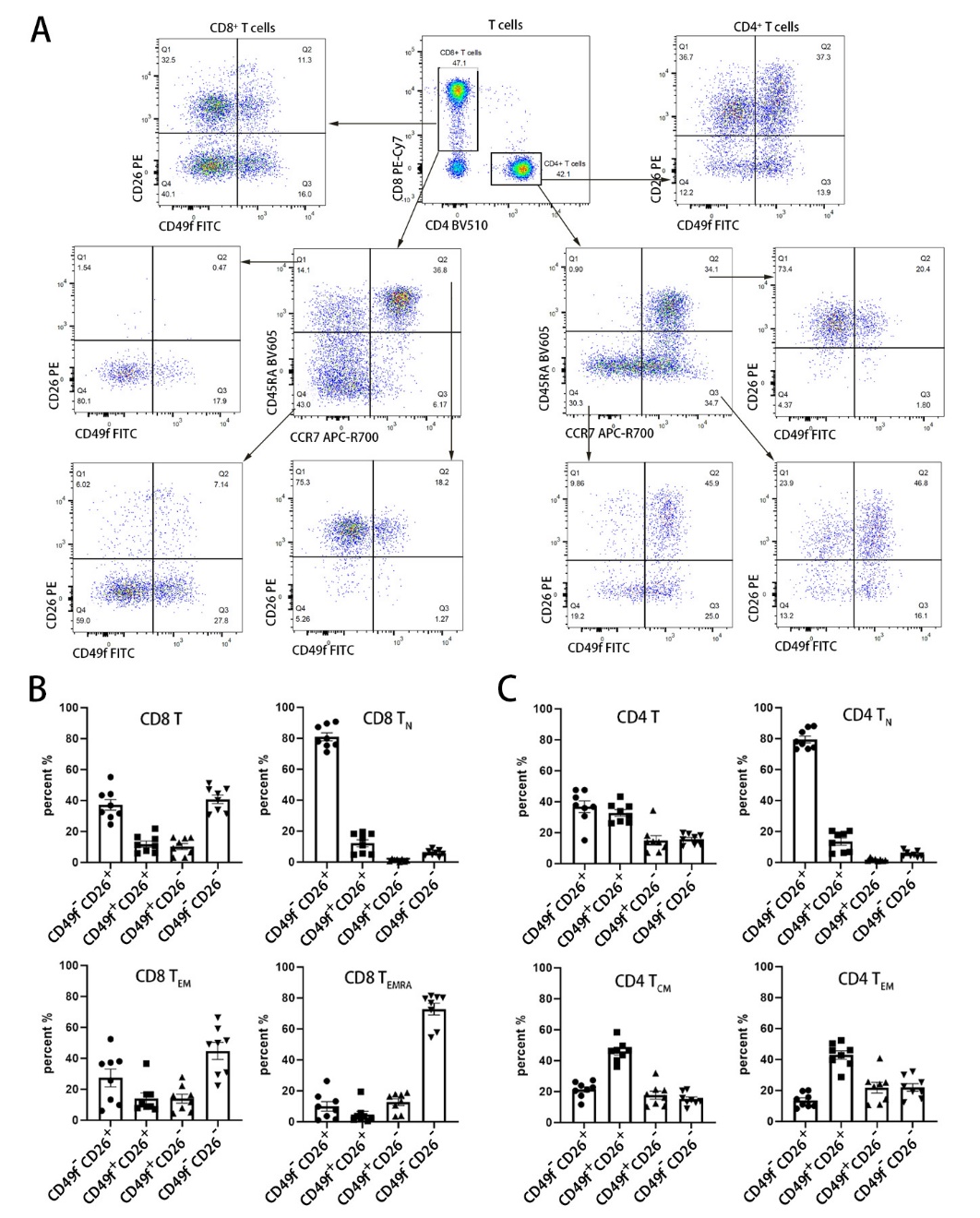
**

**Figure S3. T-cell subset subdivisions identified by CD26 and CD49f.** *A.* The expression of CD26 and CD49f in T-cell subsets identified by CD4, CD8, CD45RA, and CCR7; the arrows indicate the gating strategy to show gene expression in the corresponding subsets. Statistical information on CD26 and CD49f expression in CD8^+^ and CD4^+^ T-cell subsets is shown in *B* and *C*, respectively.

**4. Figure S4
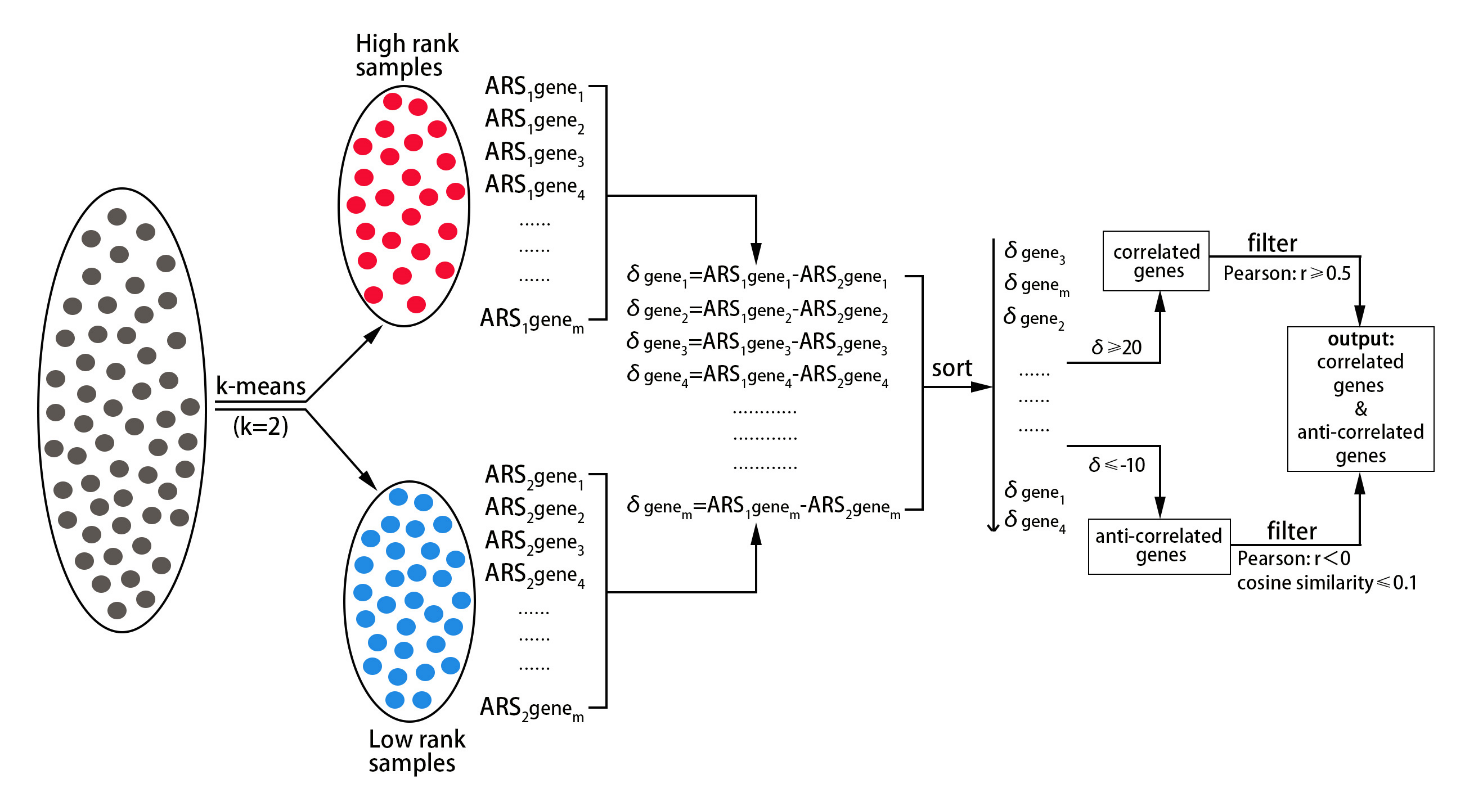
**

**Figure S4. A schematic diagram showing the virtual sorting process used in this study.** Samples were first divided into two groups by the k-means method based on the percentile rank scores of a highly plastic gene. The average rank scores (ARSs) of the same genes were recalculated in the two groups. The delta value was the difference between the ARS of the same gene in the high expression group and the ARS of the low expression group. The Delta values of all genes were sorted in descending order, and then the topmost and bottommost genes based on the indicated cutoffs were selected and further filtered by Pearson correlation and cosine similarity analyses. For RNA-Seq data, the Pearson correlation analysis used log2(TPM + 1), whereas the cosine similarity analysis directly used TPM values. Additionally, see Methods for more information.

**5. Figure S5**

**
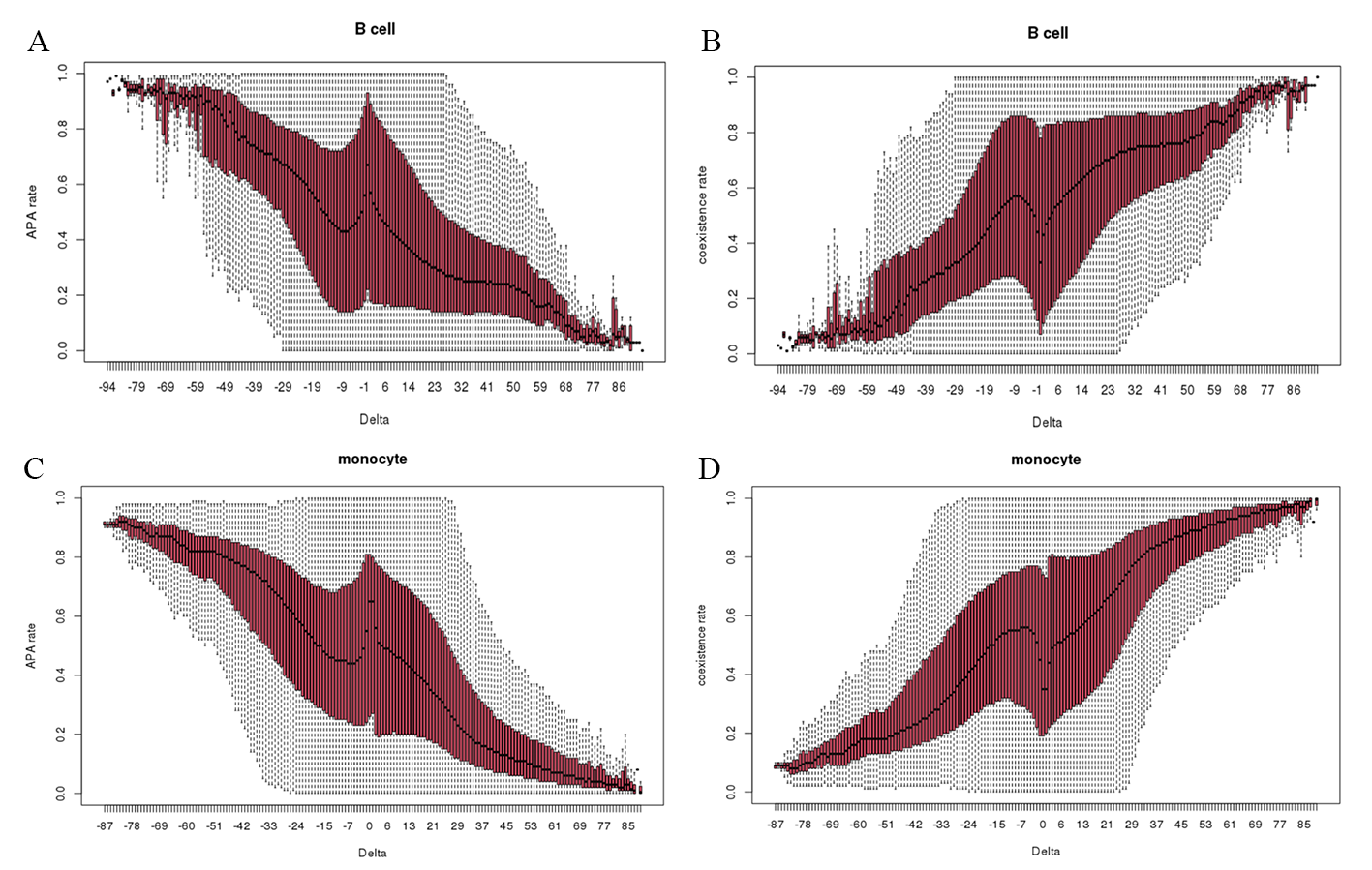
**

**Figure S5. The box plot shows the relationship between Delta and coexistence or mutually exclusive rate.** The *x*-axis in each panel indicates the Delta (δ) values from the complete virtual sorting results of 2,877 genes in B cells (*A*, *B*) and 4,183 genes in monocytes (*C*, *D*) (Table S3) based on RNA-Seq data. In *A* and *C*, the *y*-axes indicate mutually exclusive rate or APA (absent-present or present-absent in paired gene expression) rate, whereas in *B* and *D*, the *y*-axes show coexistence rate. The bottom and top of the red boxes represent the first and third quartiles, and the black band inside each box represents the median (2^nd^ quartile) of the indicated APA or coexistence rates. Different TPM cutoffs (see Methods) had similar tendencies, and the current figure represents the results from the cutoff ‘5’. Similar patterns were also observed in other cell types including CD4^+^ T cells, CD8^+^ T cells and NKs.

**6. Figure S6**


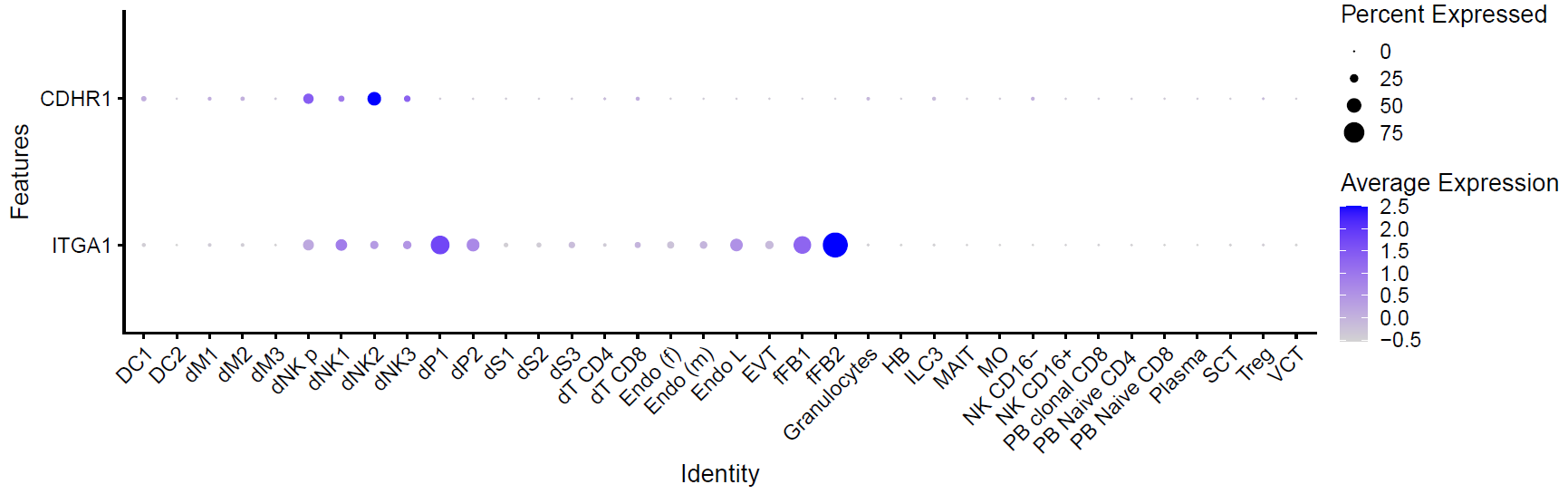

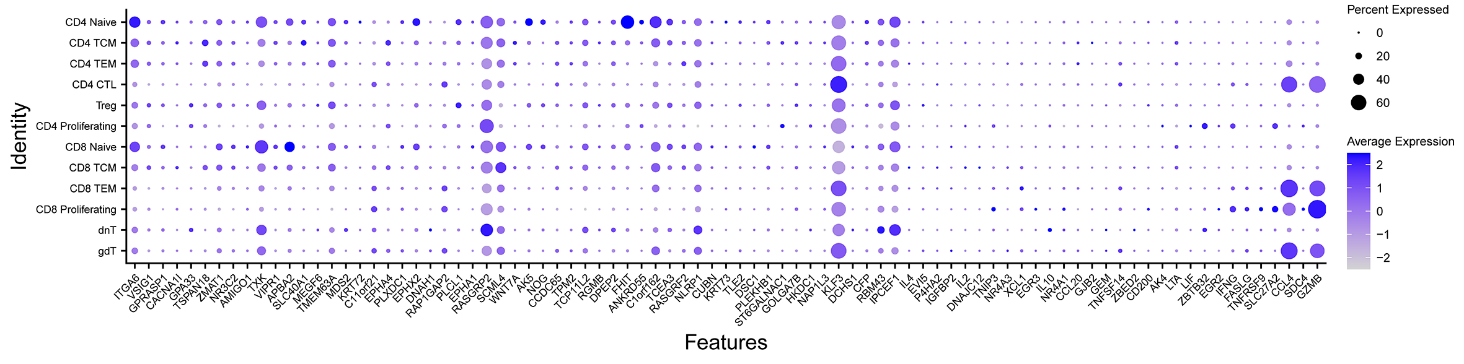


A

B

**Figure S6. The dot plot shows the expression profiles of the indicated genes in different single-cell datasets.** *A*. The correlated and anticorrelated genes of ITGA6 identified by virtual sorting in PBMCs; *B*. Differential expression of CDHR1 and ITGA1 in decidual tissue (see Figure 8 for abbreviations).

**7. Figure S7**

**
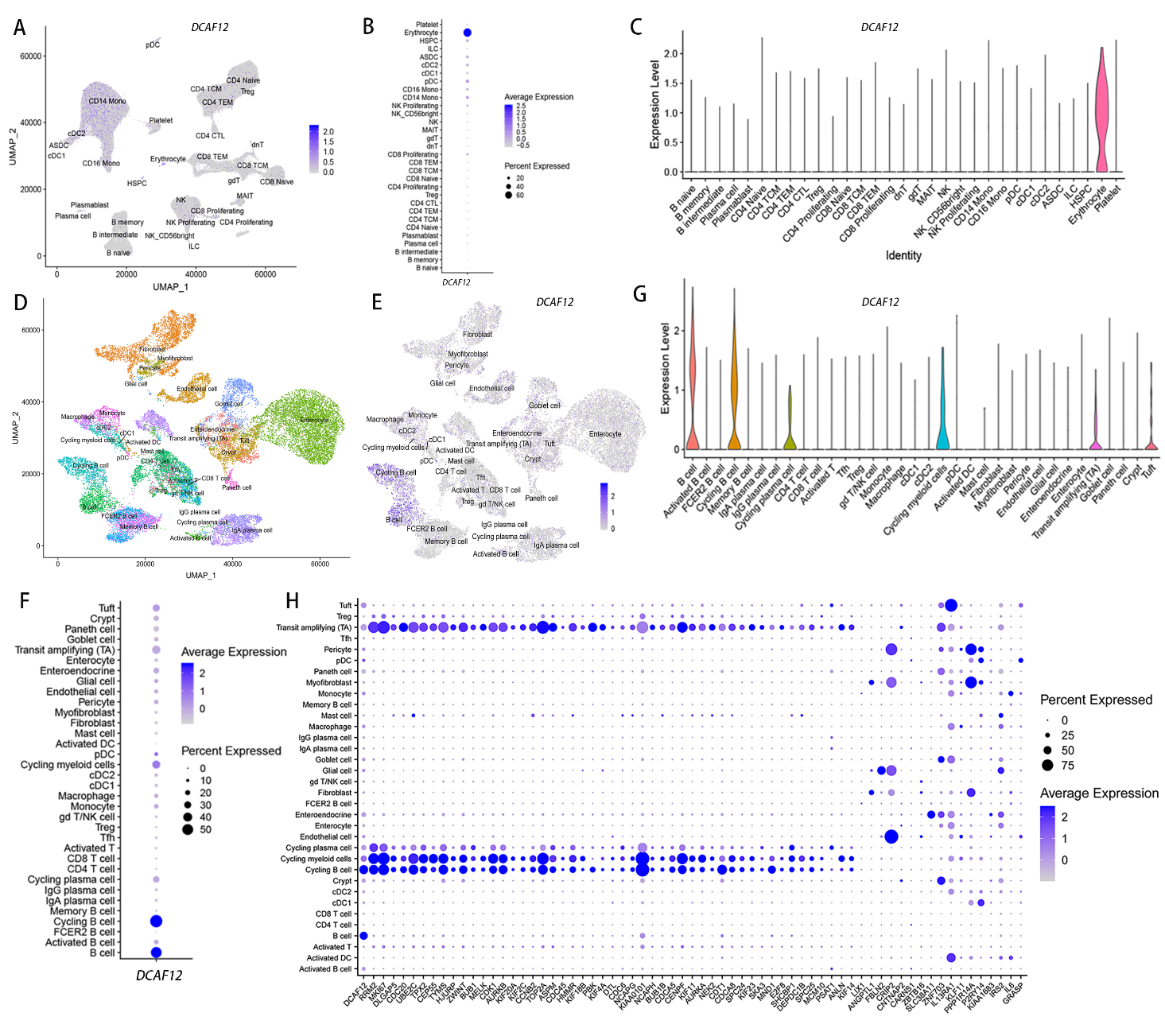
**

**Figure S7. DCAF12-positive B cells are enriched in the gut based on single-cell transcriptomic analysis.** The expression levels of DCAF12 are illustrated by feature plots (*A*, *E*), dot plots (*B*, *F*) and violin plots (*C*,*G*) in both PBMCs (*A*, *B*, *C*) and the gut (*E*, *F*,*G*). The UMAP plot in *D* is used to show cell types in the gut. The dot plot in *H* indicates the expression patterns of the correlated and anticorrelated genes of DCAF12 in gut single cells. For the correlated genes, only the top 50 genes are shown in *H*.
